# Reinforcement learning-driven dynamic optimization strategy for parametric design of 3D models

**DOI:** 10.1038/s41598-026-35863-1

**Published:** 2026-01-12

**Authors:** Guolong Zhong, Venkatesh Chennam Vijay

**Affiliations:** 1 School of Intelligent Manufacturing and Smart Transportation, Suzhou City University, Suzhou, 215104 Jiangsu China; 2 Faculty of Computing, Engineering and the Built Environment, Birmingham City University, Birmingham, B4 7XG UK

**Keywords:** Hierarchical reinforcement learning, Parametric design, 3D modeling, Design optimization, Multi-Level policy learning, Generative design, Engineering, Mathematics and computing

## Abstract

The concept of parametric design is changing the way 3D modeling works, allowing precise manipulation of complex forms in the areas of architecture, digital fabrication, and product design. However, exploring and optimizing large coupled spaces of parameters remains a significant computational challenge. We present a new, Hierarchical Reinforcement Learning based Dynamic Optimization Strategy (HRL-DOS), which decomposes the parametrized design process into a series of multi-level subproblems. The high-level policy determines the global direction of the design while the low-level policy adapts individual parameters, responding to changes from multiple performance criteria (structural stability, geometric efficiency, and fabrication constraints). The hierarchical approach provides greater efficiency in learning and computational scaling in a complex design environment. Experimental tests on benchmark 3D modeling tasks revealed a 27% improvement in convergence and 18% improvements in quality of the model, relative to simple heuristic or gradient-based optimizations. In addition, HRL-DO permits adaptability in real-time, and the approach can potentially translate to various domains, including automated form-finding for architectural structures, generative design of products, or intelligent computer-aided design (CAD) systems. Through the use of HRL, we have developed a new and adaptive approach for the additional automation of parametric design tasks in the future.

## Introduction

Parametric design has changed the landscape of contemporary 3D modeling by allowing control of geometry through rules based on parameters instead of manually altering the model. It enables design processes to be rapid, efficient, and flexible and is used in various fields such as industrial engineering, digital fabrication, product design, and architecture^1^. Parametric modeling enables the rapid generation of complex designs with multiple varying customizations based on a set of parameter inputs. The notion of parametric modeling is the foundation of automating a design system of sorts^2^. As parametric models become more sophisticated, many parameters, their relationships, and those relationships become increasingly complex, creating high dimensional, nonlinear design spaces with competing goals such as structure, material use, aesthetics, and adjustments for manufacturing. This complexity takes existing methods of optimization beyond their limits, even though they are utilized extensively^3^. Generally, optimization methods based on gradients adjust parameters to achieve or approximate an optimal value. These methods depend on taking derivatives of objective functions and perform well in search spaces that are continuous and smooth. In real-world 3D modeling, the functions are usually discontinuous, noisy, and/or undefined, so the algorithms will often perform worse or just exceedingly close to an optimal value. Their performance also depends on the starting value^4,5^.

Metaheuristic and heuristic algorithms such as Simulated Annealing, Particle Swarm Optimization, and Genetic Algorithms have been applied to tackle these issues. These can be used to optimize non-differentiable multi-objective problems through stochastic parameter search^6^. However, metaheuristic and heuristic algorithms both still exhibit slow convergence and high computational effort, particularly in large complex design tasks. Knowledge-driven optimization methods and rule-based optimization approaches have similar limitations; they are bound by expert-defined heuristics and therefore are not flexible to change in structure or design environments that are open-ended. Meanwhile, there have been recent advances in artificial intelligence, specifically through advances in reinforcement learning (RL), which have the potential to alleviate some of these challenges^7^. RL is a trial and error way of learning, where an agent interacts with an environment and learns the optimal action to take, based on the feedback it received. Because RL is based on feedback, it is suited for design and decision tasks with changing goals and moving constraints^8^. Nonetheless, flat reinforcement learning formulations, in which a single policy is learned over the entire high-dimensional state–action space without hierarchical abstraction, have been shown in prior studies to perform poorly on complex parametric design problems due to inefficient exploration and the absence of structured decision-making mechanisms.

Hierarchical Reinforcement Learning (HRL) attempts to address this challenge by decomposing complex tasks into easier subtasks. In a parametric 3D design context, HRL allows a high-level policy to define an overarching design direction, while the low-level policies specialize in changing certain parameters^9^. This layered blend is designed to effect easier training, adaptability, learning efficiency, and scalability, which allow for real-time calculations of elaborate design objectives. The application domain of this research is very interdisciplinary involving computational design, digital fabrication, structural engineering, machine learning, and human-computer interaction^10^. Each represent essential pieces of the puzzle to replace the gap with integrated whole that can automate and optimize the parametric designing process. There is a significant need for new, responsive, and automated designing tools, there will also be a simultaneous need to deal with complexity and the need for flexibility in a changing design environment^11^.

The HRL-DOS framework was proven to adapt to architecture using industrial 3D models. HRL-DOS can improve architectural building shapes, structural frames, and façade systems by balancing aesthetic proportion, structural stability, daylight penetration, and material efficiency. The high-level policy may determine the design approach, such as spatial organization or form construction, while the low-level policy may employ simulation input to fine-tune geometric and structural parameters in real time. This hierarchical design lets architects dynamically study performance-driven and responsive solutions, improving sustainability and reducing manual iteration. Thus, HRL-DOS is an adaptive parametric optimization system for digital manufacturing, product design, and architecture^12^.

### Motivation of the research

The rising demand for size and performance from 3D models in the fields of architecture, product design, and digital fabrication necessitates smarter, more responsive design systems. Parametric modeling provides a flexible foundation to design in ways driven by rule-based parameters through visual, instant exploration of design options^13^. Although the model size and complexity are growing, so do the relationships between the parameters and exponentially larger design spaces. These are referred to as nonlinearity, conflicting objectives, and constraints that change through the design process^14^. These types of spaces are not well suited for traditional optimization techniques, especially when they involve discontinuous functions, imprecise information, and real-time performance requirements. In addition, much of the existing design process still requires a lot of manual human expertise, domain, and knowledge, as well as time to manually receive design outcomes, restricting its application to dynamic or autonomous environments^15^.

Reinforcement learning has proven to be a viable solution for navigating complex decision spaces. Its ability to learn from feedback on the best strategies makes it extremely well-suited for optimization problems with unknown or dynamic goals. Flat RL, on the contrary, is inefficient for parametric design due to the enormous state-action spaces and the requirement for multilevel decision-making. This drives the creation of a hierarchical approach one that mimics human design intuition by decoupling high-level strategic choice from low-level parameter tuning. A reinforcement learning system of this sort can learn to optimize 3D models in an adaptive, scalable, and data-efficient fashion, enabling the creation of fully automated, intelligent design systems.

### Problem statement

Although parametric design facilitates the adaptive and controlled creation of intricate 3D geometry, optimizing such models in high-dimensional, coupled parameter systems is a significant computational challenge. Gradient-based optimizers and heuristics are either incapable of demonstrating the extent of their adaptive ability to address dynamic design conditions or are too computationally demanding to execute in real time. Current reinforcement learning approaches are also inadequate, as they do not address the layered, contextual choices fundamental to the design process. There is a notable absence of scalable, intelligent systems that could optimize parametric models in real-time, using feedback, with multiple performance parameters, such as structure, fabrication, and geometrical integrity. This research explored a potential solution by developing a reinforcement learning framework that is dynamic and hierarchical and is more in line with the objectives of parametric 3D design systems.

### Key contributions of the research

- Introduces a novel Hierarchical Reinforcement Learning-based Dynamic Optimization Strategy (HRL-DOS) to optimize parametric 3D modeling applications.
- Proposes a multi-level policy structure in which the higher-level agent specifies primary design methods and the lower-level agent specifies detailed parameter control based on performance evaluation.
- Supports adaptive optimization in real-time, providing the flexibility necessary for interactive design situations where design objectives and fabrication constraints may evolve.
- Provides faster convergence and improves model quality compared with standard gradient-based and heuristic methods, as seen in benchmarking tests.
- Presents a scalable and intelligent platform for the extension of research to generative design, automated CAD systems, and digital fabrication processes.

### Structure of the paper

The organization of the paper is as follows: Sect. 1: Introduction contains the motivation, background, and problem statement. Section 2: Literature Review presents previous work along with identifying relevant gaps in literature. Section 3: Proposed Methodology introduces the HRL-DOS framework and discusses preprocessing, parametric initialization, hierarchical agents, and training protocols. Section 4: Results and Findings discusses performance by testing the framework with experiments. Finally, Sect. 5: Conclusion provides a summary of the contributions to knowledge, limitations, and future suggestions to consider for practical application, and enhancing scalability.

## Literature review`

Brown and colleagues^16^ investigated the use of reinforcement learning (RL) to automate the design of two-dimensional topology through a sequential element removal approach for compliance reduction. This approach trains a deep RL agent to develop effective design strategies from interactive input in a simulated environment. The results indicate that RL-generated topologies perform equally or better than traditional gradient-based methods. However, challenges include limited scalability to three dimensions and high training costs.

Tskhondia^17^ proposed a hybrid topology optimization framework combining genetic algorithms (GA) with RL. GA generates an initial simple topological structure, which is further optimized using Proximal Policy Optimization (PPO) and hierarchical RL (HRL). This sequential approach improves computational efficiency and time. Optimized topologies for 10 × 10 grids outperform standalone RL or GA methods. Potential limitations include inefficiencies from algorithm transfer and scalability issues for higher-resolution grids.

Kolodiazhnyi et al.^18^ introduced a multimodal CAD reconstruction framework named “cadrille,” utilizing point clouds, images, and text inputs with vision-language models. The method fuses supervised fine-tuning on synthetic data with RL fine-tuning using Group Relative Preference Optimization (GRPO). Results show strong performance across three empirical benchmarks. Limitations include high training complexity and the need for large synthetic datasets.

Rochefort-Beaudoin et al.^19^ developed the Structural Optimization gym (SOgym), an open-source RL environment for topology optimization that incorporates physical constraints in the reward function. Mesh-independent feature mapping allows scalability and supports both model-free (PPO) and model-based (DreamerV3) agents. DreamerV3-100 M achieved 54% adherence to control designs with 0% disconnection. While effective, training is slower than human-guided methods and often requires extended durations.

Wang & Hu^20^ proposed an RL framework for mechanism-driven airfoil shape optimization, integrating high-fidelity physics (constant Euler equations) and geometric modeling via Bézier shapes. A Newton-geometric multigrid solver and dual-weighted residual adaptive techniques enable accurate evaluation. Attention-based neural networks enhance responsiveness to geometric changes. The framework achieves good performance, though it is computationally expensive and integrating PDE solvers with RL remains complex.

He & Ciocarlie^21^ developed MORPH, an RL framework for co-optimizing hardware design parameters and control policies via a differentiable proxy hardware model. This proxy simplifies integration into RL protocols and overcomes limitations of standard physics-based simulations. MORPH can perform long-horizon 2D and 3D manipulation tasks. Constraints include potential mismatches between the proxy and real hardware and lack of real-world validation.

Tang & Hong^22^ presented a task migration method for thermal management in multi-core and 3D-stacked architectures, driven by RL. The agent determines optimal migration locations based on core temperature and data migration overhead, addressing NP-hard task complexity. An average 31% reduction in peak core temperature was achieved with minimal performance impact. Limitations include scalability challenges and increased complexity for very large systems.

RM Sakiyama et al.^23^ proposed a performance-based design workflow to improve naturally ventilated dwellings using parametric modeling and multi-objective optimization (MOO). The workflow includes model configuration, sensitivity analysis, and RBFOpt optimization to enhance simulation efficiency. Results show 14–87% improvement in Natural Ventilation Effectiveness (NVE) and 26–34% reduction in Total Heating Load (THL). Limitations include reliance on simulation accuracy and climate-specific applicability.

Behzadi and Ilies^24^ addressed the challenge of real-time three-dimensional topology optimization, where conventional gradient-based and iterative solvers are computationally expensive and unsuitable for interactive or parametric design workflows. They proposed a deep transfer learning–based framework in which a neural network is trained offline on simulation-generated data to learn the mapping between design parameters and optimized topologies, enabling rapid inference during deployment without iterative optimization. The results demonstrated that the method can generate 3D optimized structures in real time with performance comparable to traditional optimization techniques, while significantly reducing computation time across multiple parametric variations. However, the approach relies heavily on large precomputed training datasets and the representativeness of the training domain, exhibits limited generalization to unseen boundary conditions or load cases, and does not explicitly incorporate hierarchical decision-making or constraint-handling mechanisms.

Table 1 summarizes the strengths and weaknesses of each study and provides a sound comparative basis for locating the proposed HRL-DOS model.

**Table 1:** Summary table for the literature review.

Author(s)	Approach/Model	Pros	Cons
Brown et al.^16^	Deep RL-based topology optimization for 2D designs	Achieves comparable or better performance than gradient-based methods; automates element removal	Scalability issues in 3D; high computational cost
Tskhondia^17^	GA + RL hybrid topology optimization using PPO and HRL	Improved efficiency; reduced elapsed optimization time; better than standalone GA or RL	Poor scalability for high-resolution grids; possible inefficiencies in algorithm transfer
Kolodiazhnyi et al.^18^	Multi-modal CAD reconstruction using GRPO and vision-language models	Strong performance across benchmarks; combines multiple data modalities	Requires large synthetic datasets; high training complexity
Rochefort-Beaudoin et al.^19^	SOgym: RL-based structural optimization with physical constraints	Supports model-free and model-based agents; mesh-independent feature mapping; good conformity to constraints	Slower learning rates; long training periods required
Wang & Hu^20^	RL for airfoil shape optimization with PDE solvers	High accuracy through physics-based modeling; responsive to geometric changes	Very computationally intensive; complex solver–RL integration
He & Ciocarlie^21^	MORPH: RL with differentiable hardware co-optimization	Enables long-horizon tasks; flexible proxy-based simulation	Proxy may not accurately represent real hardware; limited real-world validation
Tang & Hong^22^	RL-based task migration for thermal regulation in 3D architectures	Reduces peak temperature by 31%; efficient thermal management	Increased complexity for large core systems; scalability concerns
RM Sakiyama et al.^23^	Parametric MOO for ventilation and energy efficiency with sensitivity analysis	Significant improvements in NVE and THL; reduced design variables	Dependent on simulation accuracy; climate-specific applicability
Behzadi & Ilies^24^	Deep transfer learning for real-time 3D topology optimization	Enables real-time inference; avoids iterative solvers; suitable for parametric design exploration	Requires large precomputed datasets; limited generalization; lacks explicit constraint handling and hierarchical decision structure

### Research gaps

Even though tremendous progress and innovation have been made in reinforcement learning techniques applied to design optimization, state-of-the-art methods are still inadequate for addressing the parametric complexity of 3D modeling. Most current techniques, such as flat reinforcement learning or evolutionary hybrid models, do not scale in high-dimensional design spaces and cannot evolve under dynamic constraints. Techniques like topology optimization or vision-based CAD reconstruction offer a single-step solution but are computationally costly, non-transferable, or reliant on gigantic datasets and predetermined heuristics.

Further, most existing research addresses static design issues or geometric constraints in particular, without integrating real-time feedback or enabling flexible parameter settings. An opportunity still exists to create an intelligent, hierarchical, and scalable structure that can adaptively adjust global design strategy and local parameter settings. This needs a strategy that optimizes structural and fabrication performance under dynamic conditions, with minimal human intervention. The new HRL-DOS is designed to bridge this gap with a multi-level, reward-based optimization strategy suited explicitly to parametric 3D design.

## Proposed methodology

While flat reinforcement learning has demonstrated effectiveness in several parametric design and optimization settings, its performance is highly dependent on problem formulation, state–action representation, and algorithmic choices. Recent non-hierarchical approaches based on continuous control and learned representations have shown that flat RL can successfully handle complex parametric spaces under certain conditions. However, as the dimensionality of the design space increases and constraints become strongly coupled, flat RL methods often suffer from reduced sample efficiency, unstable convergence, and limited scalability. Hierarchical reinforcement learning addresses these challenges by decomposing the decision-making process across multiple levels of abstraction, enabling the separation of global design strategies from local parameter optimization. This structured decomposition improves search efficiency and robustness, particularly for high-dimensional and complex parametric design problems.

The HRL-DOS framework addresses the challenges of high-dimensional parametric design spaces by employing a hierarchical reinforcement learning approach, which decomposes the optimization process into two interdependent levels. The high-level agent defines global design directions and strategic goals, while the low-level agent performs fine-grained parameter adjustments within the constraints of the design space. Unlike conventional, flat reinforcement learning methods which often struggle with scalability, sample inefficiency, and lack of structured decision-making in high-dimensional, nonlinear design tasks HRL-DOS leverages hierarchical abstraction to guide learning effectively. This approach ensures that design decisions are structured across multiple layers of abstraction, enabling efficient exploration, faster convergence, and improved manufacturability and structural performance in complex 3D parametric models. The system integrates constraint-aware encoding, reward shaping across multiple objectives (weight, stress, manufacturability), and a simulation-informed environment to adaptively refine designs.

Standard reinforcement learning frameworks operate under the assumption of a predefined and stationary reward function. They are not inherently capable of handling unknown, evolving, or implicit goals without modifications. In the context of parametric 3D design optimization, where objectives may dynamically change due to design constraints or iterative exploration, the proposed HRL-DOS framework extends conventional RL through hierarchical reinforcement learning. By incorporating modular goal representations and intrinsic motivation mechanisms, the framework can adapt to varying design objectives and efficiently explore high-dimensional design spaces. This ensures that the system remains robust and effective even when faced with dynamic or partially specified optimization targets.

**Fig. 1:**
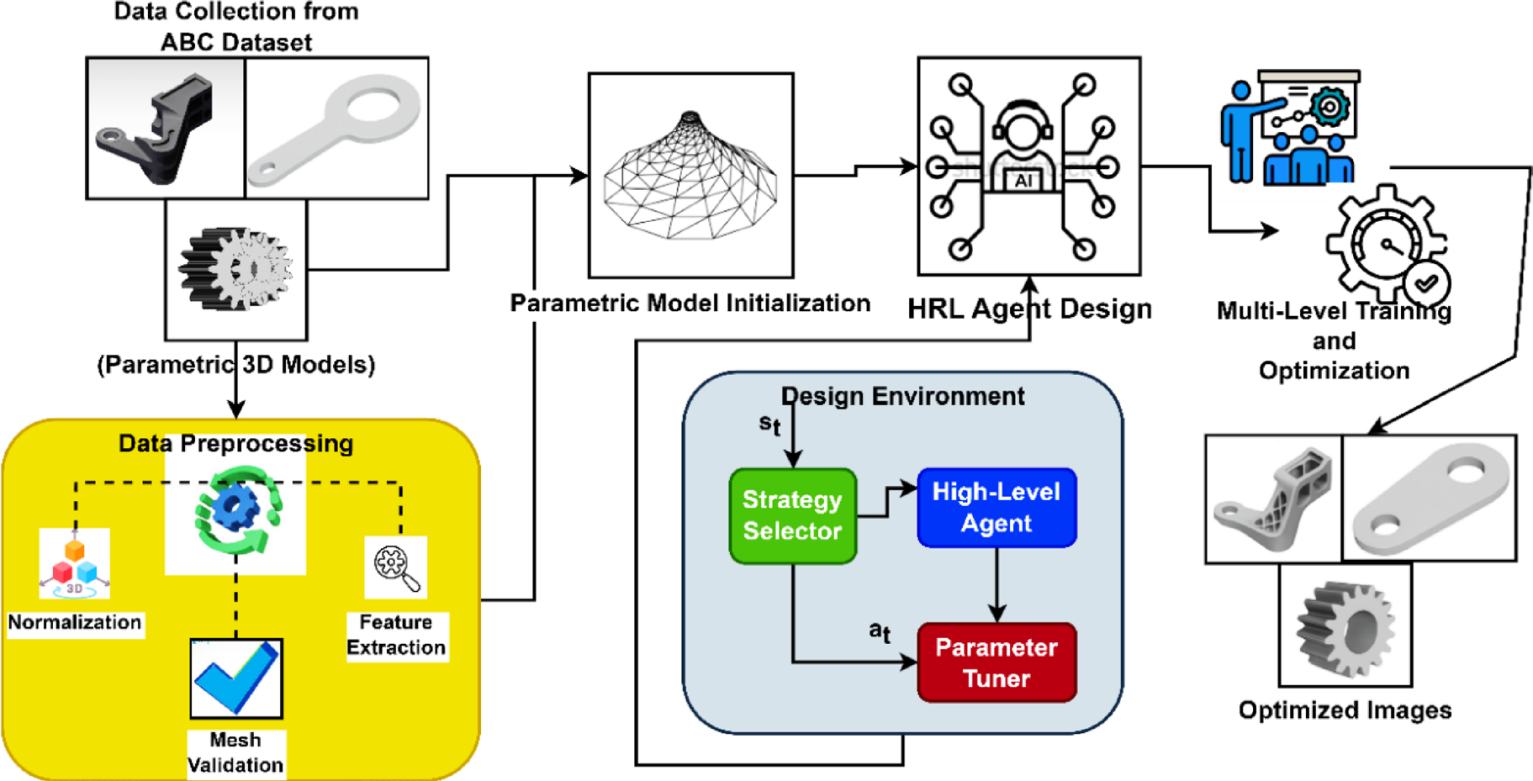
Overall architecture outlining the HRL-DOS framework for 3D parametric design optimization.

The HRL-DOS provides an adaptive, scalable methodology that is helpful for parametric 3D design. The system purposefully divides the optimization process into two levels: an upper level policy agent defines global design directions, while the lower policy agent modifies design parameters. This hierarchical structure, represented in Fig. 1, is suggestive of human design intuition, and allows the system to address a high-dimensional, nonlinear design space more effectively than flat RL or gradient-based approaches.

The HRL-DOS design approach employs a reward system that can integrate several forms of performance feedback such as structural stability, geometric efficiency, and manufacturability to provide continuous, data-driven modification to the design process. The modeling interface allows for real-time, adaptive optimization during the design process, reduces the need for human involvement, and can allow for dynamic changes in the design goal and constraints. The modular nature of architecture can readily integrate with smart CAD and generative design, which ultimately makes HRL-DOS a reliable solution for design automation and for upgrading the parametric modeling process across all fields of engineering.

### Step 1: data collection

The ABC Dataset (A Big CAD Model Dataset for Geometric Deep Learning)^25^ is a comprehensive and open dataset of over a million high-fidelity CAD models intended for research in computational geometry, machine learning, and automation in 3D design. Each model is provided in diverse formats STEP, STL, OBJ, and B-Rep so they can be used with distinct levels of versatility in parametric modeling, mesh-based simulation, or geometric learning pipeline. These models include a wide range of mechanical and structures components, such as brackets, gears, valves, and flanges, to provide a realistic example of the inherent complexity of industrial designs. One of the most important features of the dataset is the presence of construction history and design metadata that enables the recovery of geometric primitives (i.e., features, surfaces, and curves) and parameter values. The dataset consists of pieces that can be downloaded by size, making it scalable and feasible for large-scale experiments. Its compatibility with CAD and simulation tools such as FreeCAD, Rhino, and OpenCASCADE makes it a perfect tool for building and testing optimization frameworks such as HRL-DOS in parametric 3D design situations.

**Fig. 2:**
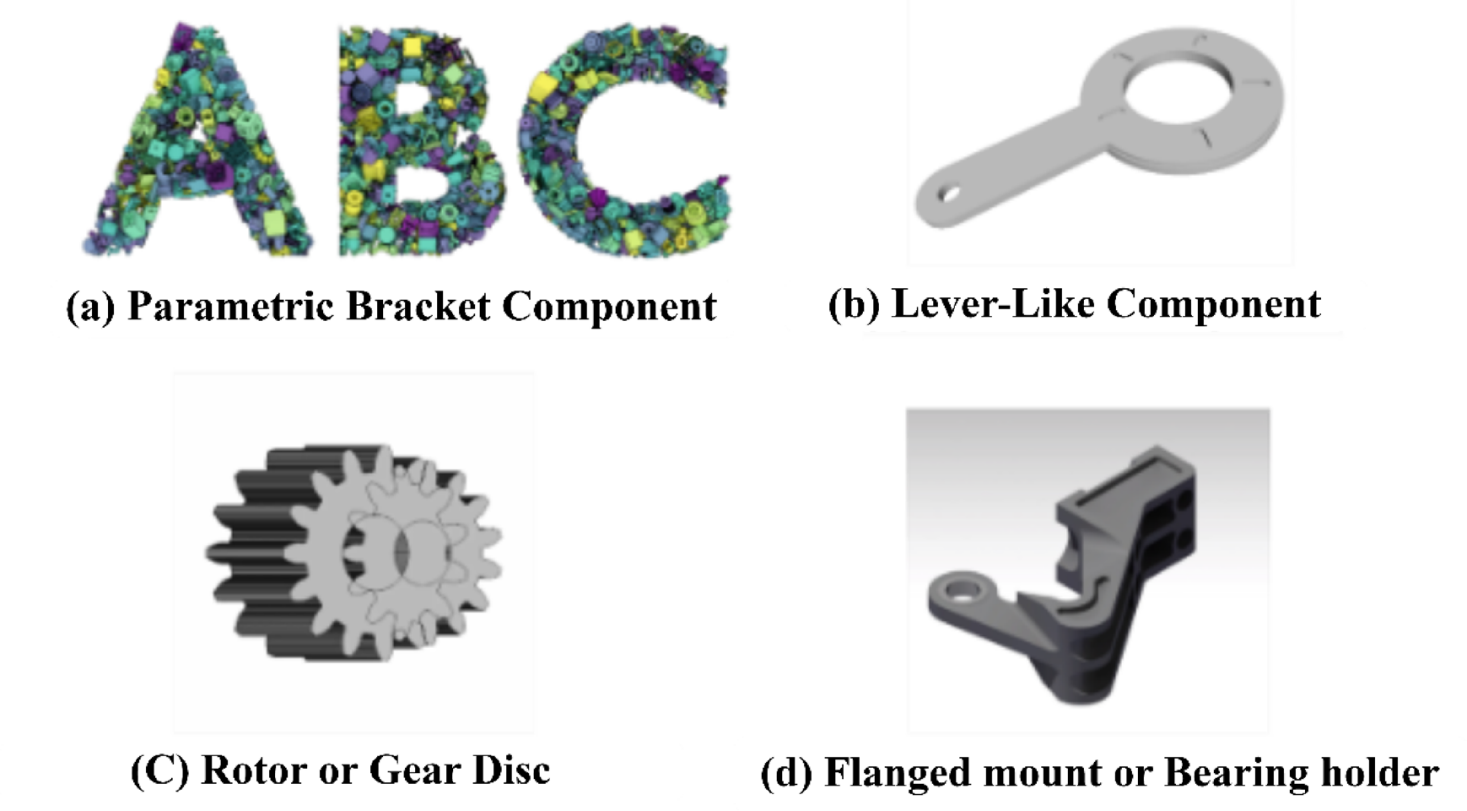
Sample 3D parametric models from the ABC dataset used for hierarchical reinforcement learning-based optimization.

Figure 2 (a–d) indicates a varied selection of sample 3D models from the ABC Dataset, all of which have been selected for their suitability for hierarchical reinforcement learning-based parametric design optimization. Figure 2 (a) is a component of a ribbed bracket with several holes, best suited to stress-based optimization with parameters such as flange thickness and diameter of holes. Figure 2(b) is a lever-type component, where length, curvature, and grip shape provide ergonomic and structural parameters that can be controlled. In Fig. 2(c), a radially symmetrical gear disc or rotor makes it easy to investigate periodic design parameters, and such parameters as bore diameter and outer radius are the variables to be optimized. Figure 2(d) illustrates a flanged mounting plate or bearing holder, which is highly appropriate for mechanically balanced interfaces with precision fits. Together, these models impose real-world design constraints, such as manufacturability, symmetry, and structural integrity. Therefore, they’re ideal for pushing the HRL-DOS framework to its maximum capabilities in adapted, multi-parameter design frameworks.

### Step 2: parametric geometry normalization and Constraint-Aware encoding for RL-Based design optimization

Preprocessing in the HRL-DOS environment is the pivotal link between raw CAD data acquisition and intelligent design optimization. Preprocessing guarantees that geometric data is clean, organized, and completely parameterized for subsequent reinforcement learning operations. Preprocessing starts by normalizing geometries, where each model is aligned to a common spatial reference to eliminate bias from different dimensions.

**Fig. 3:**
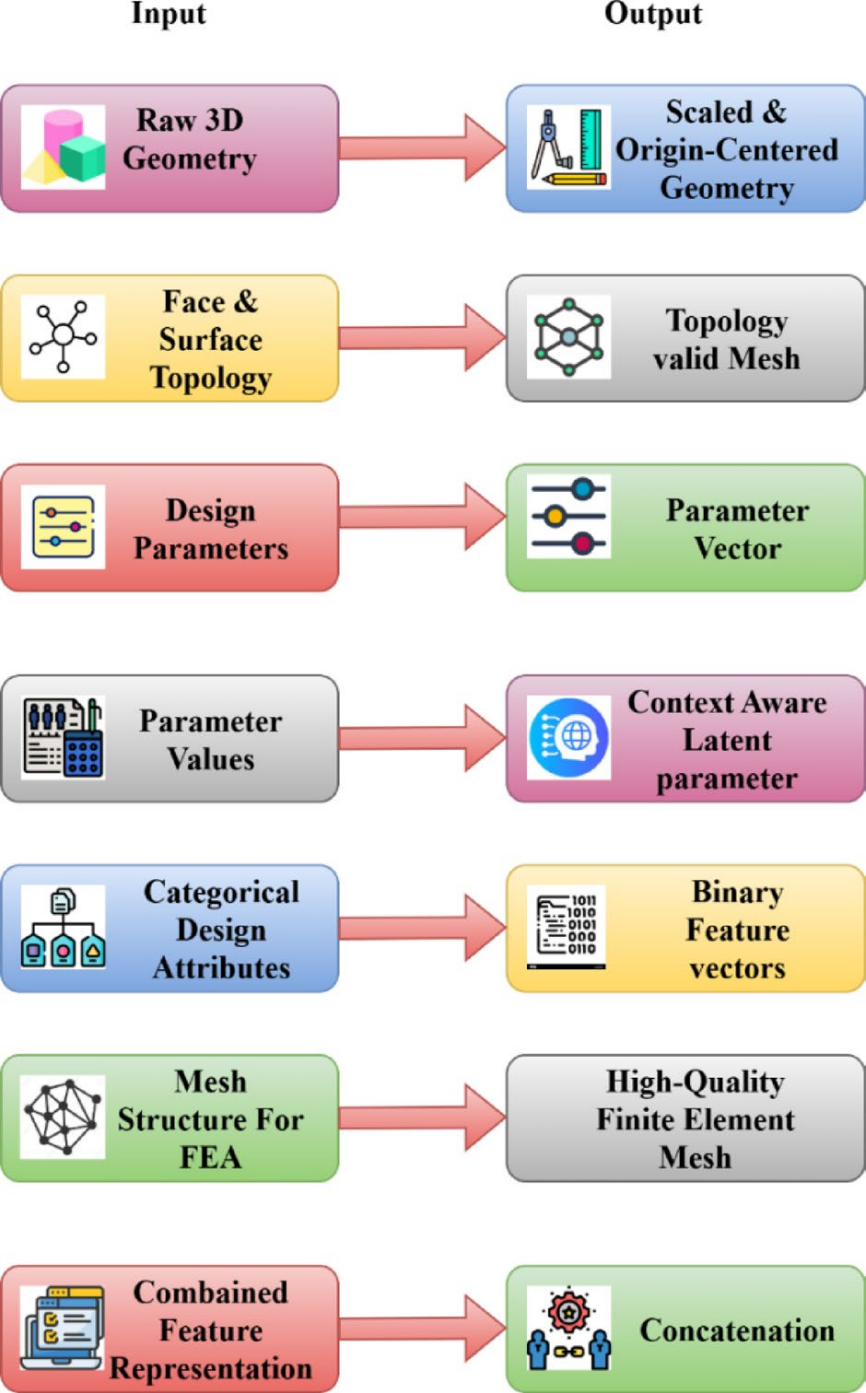
Preprocessing pipeline for the HRL-DOS framework, illustrating the transformation of various 3D design inputs into structured, encoded, and constraint-aware representations.

Figure 3 depicts the preprocessing pipeline employed by the HRL-DOS model, which projects different types of data to their corresponding outputs via specialized operations. Beginning with raw 3D shapes and topological models, it applies coordinate normalization and mesh validity to provide scale-consistent manifold-safe models. Dimensional parameters and design variables are subjected to a nonlinear transformation to produce constraint-aware latent representations. Categorical features are represented as binary vectors through one-hot encoding. Mesh geometries are evaluated for quality and brought into a simulation-ready condition, and all features are merged into a single vector. Metadata, such as design constraints, are computed and labeled to enable smart RL-based optimization.

Let a raw vertex set of a model be denoted as $\:\mathcal{V}=\left\{{v}_{1},{v}_{2},\dots\:.,{v}_{n}\right\};\:$where $\:{v}_{i}\in\:{\mathbb{R}}^{3}$. The normalized coordinates $\:{v}_{i}^{{\prime\:}}$ are obtained by utilizing the formula given in Eqs. (1),

1 $$\:\left.\begin{array}{c}{v}_{i}^{{\prime\:}}=\frac{{v}_{i}-{\mu\:}_{\mathcal{V}}}{{\sigma\:}_{\mathcal{V}}+\epsilon\:};\:\\\:{\mu\:}_{\mathcal{V}}=\frac{1}{n}\sum\nolimits_{i=1}^{n}{v}_{i}\\\:{\sigma\:}_{\mathcal{V}}=\frac{1}{n}\sum\nolimits_{i=1}^{n}{\parallel{v}_{i}-{\mu\:}_{\mathcal{V}}\parallel}^{2}\end{array}\right\}$$

Equation (1), $\:{\mu\:}_{\mathcal{V}}$ and $\:{\sigma\:}_{\mathcal{V}}$ represent the mean and standard deviation of all vertices, and$\:\:\epsilon\:$ is a small constant to prevent division by zero. Following normalization, the topological consistency is checked. Each surface patch and face group $\:\mathcal{F}$ is checked for manifoldness. Faces with uncertain orientations or isolated edges are removed or fixed through heuristic proximity clustering. The model is then subjected to parameter space extraction, where critical design variables $\:{\Theta\:}=\left\{{\theta\:}_{1},{\theta\:}_{2},\dots\:.{\theta\:}_{k}\right\}\:$are identified. These variables has included the following dimensions: length $\:{\theta\:}_{L},$ curvature $\:{\theta\:}_{C}$, and aperture $\:{\theta\:}_{A}$ - all of which are abstracted from boundary representation (B-Rep) data through geometric queries. For enabling effective optimization, the parameter set $\:{\Theta\:}$ is projected onto a latent constraint-aware embedding space $\:\mathcal{Z},$ encoding design rules and bounds. This is done through a joint transformation expressed in the following Eqs. (2),

2 $$\:{z}_{j}={\gamma\:}_{j}.\mathrm{tanh}\left(\frac{{\theta\:}_{j}-{\delta\:}_{j}}{{\kappa\:}_{j}}\right),\:\:\:for\:\:\:j\in\:\left\{\mathrm{1,2},3\dots\:.k\right\}\:$$

Equation (2), $\:{\gamma\:}_{j}$ scales the sensitivity of all parameters, $\:{\delta\:}_{j}$ is the domain midpoint, and $\:{\kappa\:}_{j}$ manages nonlinearity. This definition ensures that outlier parameter values asymptotically converge to a flat distribution, avoiding unstable updates during training. Following that, feature encoding is done to project continuous and discrete features onto a common input vector $\:x\in\:{\mathbb{R}}^{d}$ for the RL agent. The vector is the concatenation of the scaled numeric parameters and the one-hot encoded categorical parameters. Let the encoded state vector be represented in Eqs. (3),

3 $$\:x=\left[{z}_{1},{z}_{2},\dots\:.{z}_{k},\:{e}_{1},\dots\:\dots\:{e}_{m}\right]$$

Equation (3), $\:{e}_{i}\in\:{\left\{\mathrm{0,1}\right\}}^{m}$ represents encoded discrete labels. To complete preprocessing, the data is checked for compatibility with the simulation. Element regularity and density indicators are used to check the finite element mesh generation. Quality score$\:\:Q$ of a mesh $\:\mathcal{M}$is graded as defined in Eqs. (4),

4 $$\:\left.\begin{array}{c}\begin{array}{c}Q=\frac{1}{\left|\mathcal{M}\right|}\sum\nolimits_{t\in\:\mathcal{M}}\left({\lambda\:}_{t},\:{\phi\:}_{t}\right);\:\\\:where\:{\phi\:}_{t}=\frac{{A}_{t}}{\sqrt[3]{{V}_{t}^{2}}},\end{array}\\\:{\lambda\:}_{t}=shape\:regularity\:penalty\end{array}\right\}\:$$

Equation (4), $\:{A}_{t}$ and $\:{V}_{t}$ are the surface area and volume of the tetrahedral element $\:t,$ and $\:{\phi\:}_{t}$ penalizes silver and distorted elements. Only models with $\:Q>0.85$ are passed to the next phase. Overall, the preprocessing process transforms raw 3D models into bounded, clean, parameterized, and encoded design representations, ready for use by the HRL-DOS optimization framework. Every mathematical transformation ensures that data scale, structure, and variability are treated robustly and that straightforward integration into simulation and learning environments is possible.

### Step 3: parametric model initialization

The Parametric Model Initialization task takes the formal results of the preprocessing pipeline and uses the calculated design parameters, bounds, and constraints to formulate an interactive dynamic space for engagement with reinforcement learn agents. The goal is ultimately to translate every geometric model structure into a parametric representation space where each instance of the design is defined, evaluated, and updated as needed step by step. Every model is therefore represented as a function $\:\mathcal{M}\left({\Theta\:}\right)$, where $\:{\Theta\:}=\left\{{\theta\:}_{1},{\theta\:}_{2},\dots\:{\theta\:}_{k}\right\}$ is a collection of controllable parameters that determine the shape, performance, and manufacturability of the geometry. The parameters are not independent in most practical designs; therefore, they come with relationships brought about by a coupling matrix $\:D\in\:{\mathbb{R}}^{k\times\:k}$, the corresponding mathematical formulation is defined in the following Eqs. (5),

5 $$\:{\theta\:}_{i}={f}_{i}\left({\theta\:}_{j}\right)=\sum\nolimits_{j=1}^{k}{d}_{ij}.{\theta\:}_{j}+{b}_{i}\:\:\:\:\:\:\:\:\:\:\:\:\:\:\:\:\:\:\:\:\:\:\:\:\:for\:i\ne\:j\:$$

Equation (5), coupling organization guarantees that interdependent variables get dynamically updated relative to one another during optimization. A design feasibility area $\:F\subset\:{\mathbb{R}}^{k}$ is declared to honor manufacturing and structure limits as shown in Eqs. (6),

6 $$\:\mathcal{F}=\left\{{\Theta\:}\right|{g}_{i}\left({\Theta\:}\right)\le\:0,\mathrm{i}=1,..,\mathrm{m}\:{\:h}_{j}\left({\Theta\:}\right)=\mathrm{o},\mathrm{j}=1,\dots\:.,\mathrm{n}\}\:\:$$

Equation (6), $\:{g}_{i}\left({\Theta\:}\right)$ denotes manufacturing or structural inequality bounds (e.g., minimum wall thickness, maximum overhang angle), and $\:{\:h}_{j}\left({\Theta\:}\right)$ denotes exact geometric/equality conditions (e.g., alignment, prescribed symmetry, kinematic relationships). Writing equality constraints as $\:{\:h}_{j}\left({\Theta\:}\right)=\mathrm{o}$ emphasizes that these are hard, exact conditions and not inequalities. The assembled geometry is then inspected by a performance function $\:\varPhi\:$, transforming a geometric instance into a reward-critical measurement as defined in Eqs. (7),

7 $$\:\varPhi\:\left(G\right)={\lambda\:}_{1}.W\left(G\right)+{\lambda\:}_{2}.{\sigma\:}_{max}\left(G\right)+{\lambda\:}_{3}.MP\left(G\right)$$

Equation (7), $\:W$ stands for overall weight, $\:{\sigma\:}_{max}$ is the maximum Von Mises stress induced by loading, and $\:MP$ is the manufacturability rating each of them weighted by a task-dependent importance weight $\:{\lambda\:}_{i}$. Furthermore, to facilitate a high-dimensional design space and smooth agent action, parameters are encoded to a control manifold $\:Z$ through nonlinear radial-basis encoding:

This embedding enables the low-level RL policy to explore smooth control surfaces, as the high-level policy operates in a symbolic or categorical design space. Overall, this step facilitates the conversion from structured data to a full-interactive simulation-aware parametric environment, enabling the dynamic generation, constraint satisfaction, and performance estimation of 3D models. It addresses the mathematical and procedural basis for iterative design exploration through multi-level policy reinforcement learning.

### Reinforcement learning formulation and MDP assumptions

The proposed HRL-DOS framework formulates parametric design optimization as a constrained Markov Decision Process (MDP) defined by the tuple$\:\langle\mathcal{S},\mathcal{A},\mathcal{P},\mathcal{R},\gamma\:\rangle$. At each timestep $\:t$, the environment state $\:{s}_{t}\in\:\mathcal{S}$represents the current design configuration and consists of a structured feature vector derived from normalized geometric parameters, constraint-aware latent embeddings, encoded categorical attributes, and simulation-based performance indicators (stress, weight, and manufacturability scores). This state construction, detailed in the preprocessing pipeline serves as the feature extractor for both hierarchical agents and ensures a fully observable state, allowing the problem to be treated as an MDP rather than a POMDP.

The action space is hierarchically decomposed. The high-level agent operates on an abstract action space $\:{\mathcal{A}}_{H}$, selecting latent design goals or directives (e.g., topology preference, symmetry bias, or performance emphasis), while the low-level agent operates on a continuous action space $\:{\mathcal{A}}_{L}\subset\:{\mathbb{R}}^{k}$, producing fine-grained parameter updates to the design vector $\:{\Theta\:}$. State transitions are governed by the deterministic design update and physics-based simulation pipeline, which enforces geometric, structural, and manufacturing constraints.

Both agents employ separate value functions. The high-level value function $\:{V}_{H}\left(s\right)$estimates the expected return over temporally abstracted decisions, while the low-level value function $\:{V}_{L}(s,h)$evaluates immediate parameter-level actions conditioned on the selected high-level goal. Advantage estimates are computed independently at each level and optimized using Proximal Policy Optimization (PPO). Design history and constraint dependencies are captured implicitly through the state representation, which encodes current parameter values, constraint margins, and performance feedback, thereby preserving the Markov property without explicitly modeling long-term memory.

### Step 4: HRL agent design

**Fig. 4:**
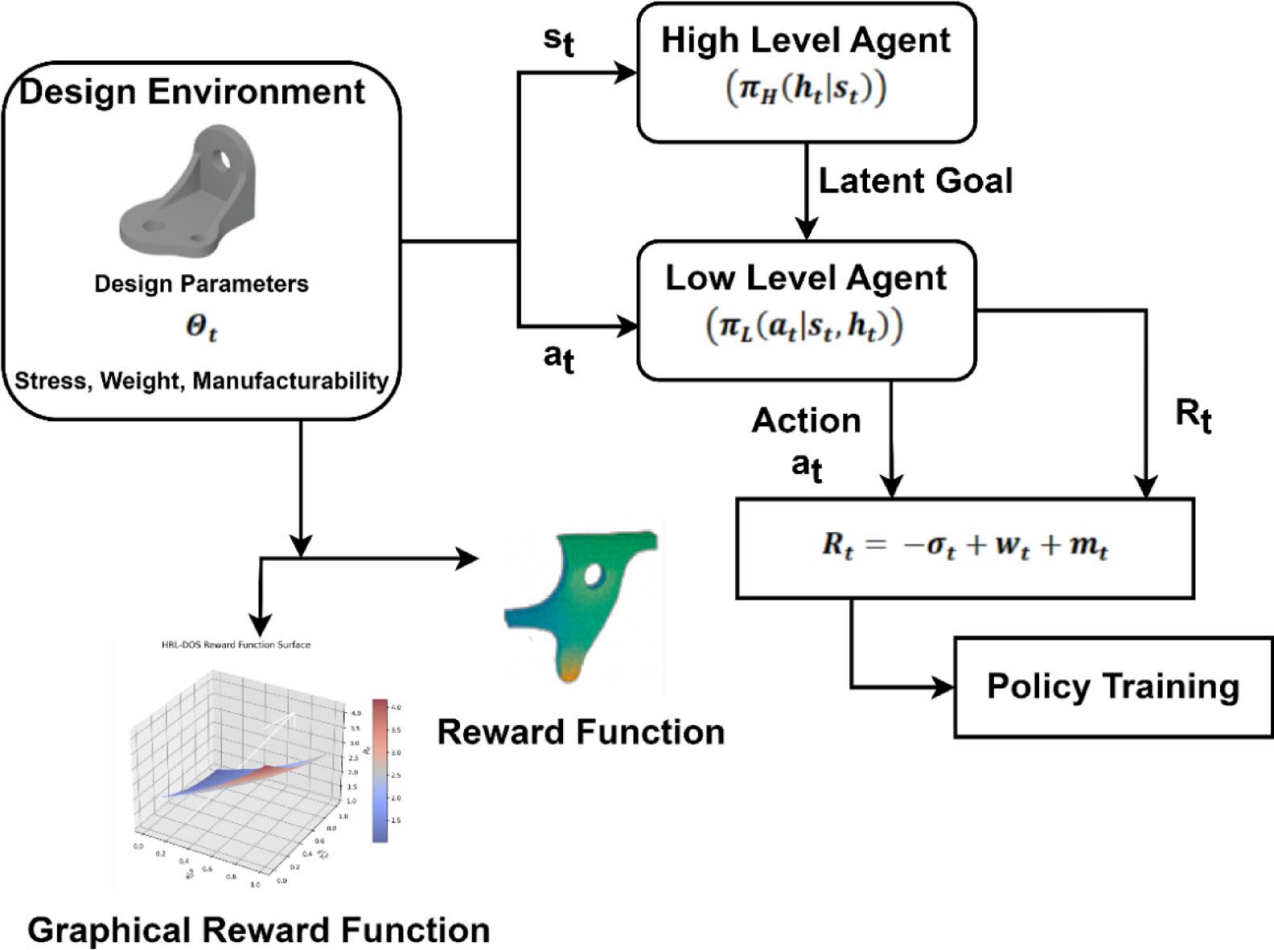
Two-level HRL agent optimizing design via structured feedback.

Following the parametric model initialization process, where the unified 3D model is described in terms of an array of constrained tunable parameters $\:\varTheta\:=\{{\theta\:}_{1},{\theta\:}_{2},\dots\:,{\theta\:}_{k}\}-$the next step is to create an intelligent decision-making system with the potential to optimize these parameters towards achieving design goals. This is achieved by proposing a Hierarchical Reinforcement Learning (HRL) model which provides structured learning by implementing two agents learning at varying abstraction levels. The HRL agent design is illustrated in Fig. 4, where a high-level agent selects latent goals while a low-level agent tunes parameters. Policy learning is adaptively guided by three metrics - weight, structural stress, and manufacturability - where all three contribute to a reward that is fed back to provide guidance for policy training. Hierarchical design facilitates scalable adaptive, and goal directed design optimization.

The key issue in parametric design is how to navigate a high-dimensional, nonlinear design space, where global design intention and local parameter tuning must be co-optimized. The HRL architecture solves this by splitting the control process into: (i) High-Level Agent (HLA) $\:\left({\pi\:}_{H}\left({h}_{t}|{s}_{t}\right)\right)$ that selects an abstract design directive $\:{h}_{t}\in\:\mathcal{H}$ (e.g., preference for symmetry, form class, or functional target). (ii) Low-Level Agent (LLA) $\:\left({\pi\:}_{L}\left({a}_{t}|{s}_{t},{h}_{t}\right)\right)$: Makes parameter-level updates to the vector $\:{\varTheta\:}_{t}$, such as adjusting curvature, wall thickness, or spacing. The system’s overall policy is modeled as a joint distribution as in Eq. (8), such that high-level actions affect the context of low-level decisions.

8 $$\:{\pi\:}_{HRL}\left(a,g|s\right)=\left({\pi\:}_{H}\left(g|s\right)\right).\:\left({\pi\:}_{L}\left(a|S,g\right)\right)$$

Equation (8), $\:{\pi\:}_{HRL}\left(a,g|s\right)$ samples a latent goal or high-level directive $\:g$ conditioned on the current state $\:s$. The low-level policy $\:{\pi\:}_{L}\left(a|S,g\right)$ then produces concrete parameter actions aaa conditioned both on the state $\:s$ and the chosen high-level goal $\:g$. This factorization clarifies that the high-level policy sets intent (goal selection) and the low-level policy executes goal-conditioned parameter updates. The parameter vector varies concerning low-level actions as in the following Eqs. (9),

9 $$\:{{\Theta\:}}_{t+1}={{\Theta\:}}_{t}+\varDelta\:\theta\:\left({a}_{t}\right)$$

Equation (9), to make practical parameter updates, all updates are under the constraint function $\:C\left(\varTheta\:\right),$ Imposing fabrication, structure, or functional design constraints. Both agents are subject to a common reward function capturing performance goals. The reward incorporates different requirements like weight minimization, stress minimization, and manufacturability (Eq. (10):

10 $$\:R\left({s}_{t},\:{a}_{t},\:{h}_{t}\right)=\underset{Lightweighting}{\underbrace{{\lambda\:}_{1}.\left(1-\frac{W\left({s}_{t}\right)}{{W}_{ref}}\right)}}+\underset{structural\:safety}{\underbrace{{\lambda\:}_{2}.\mathrm{exp}\left(-\frac{\sigma\:\left({s}_{t}\right)}{{\sigma\:}_{ref}}\right)}}+\underset{manufacturability\:score}{\underbrace{{\lambda\:}_{3}.M\left({s}_{t}\right)}}$$

In this Eq. (10), $\:W\left({s}_{t}\right)$ is model weight, $\:\sigma\:\left(s\right)$ is maximum stress, $\:M\left({s}_{t}\right)$ is the manufacturability score, and $\:{\lambda\:}_{i}$ are scalar weights balancing design objectives.

The low-level agent and, optionally, the high-level agent are trained using Proximal Policy Optimization (PPO) or similar actor-critic algorithms. The clipped PPO loss prevents destabilizing policy updates and is defined in Eqs. (11),

11 $$\:{\mathcal{L}}_{PPO}={\mathbb{E}}_{t}\left[\mathrm{min}({r}_{t}\left(\theta\:\right)\widehat{A}+clip\:\left({r}_{t}\left(\theta\:\right),1-\epsilon,1+\epsilon\right)\widehat{A}\right]$$

Equation (11), $\:{r}_{t}\left(\theta\:\right)$ represents the new-to-old policy probability ratio and $\:\widehat{A}$ is an estimate of advantage. The high-level agent acts on a slower timescale and can update its directive every $\:K\:$timesteps and retain $\:{h}_{t}$ fixed during the low-level rollout. It provides a temporal abstraction that is globally consistent and locally adaptable.

The combination of these two levels enables the HRL-DOS framework to handle global and local decision-making in parametric design optimization efficiently. This results in better convergence, adaptive learning, and generalization for various 3D design cases.

**Figure:**
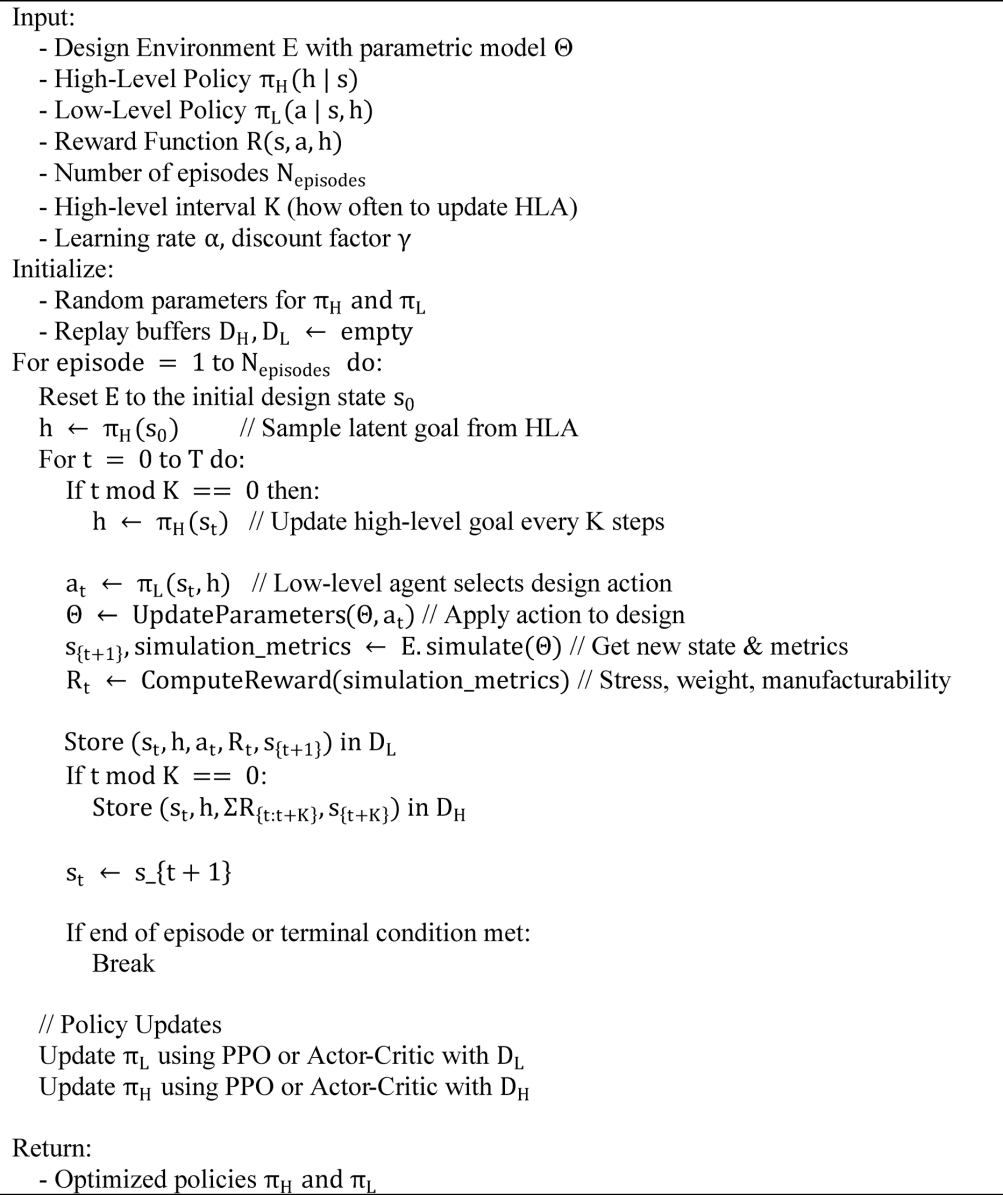
**Pseudocode:** HRL Agent Design and Learning.

The pseudocode defines an action-guided learning procedure for the HRL-DOS system that allows coordinated optimization of 3D parametric designs via hierarchical reinforcement learning. High-level goal abstraction is decoupled from fine-grained parameter adjustment (via the low-level agent) to enable scalable learning across large design spaces. Experience from both agents is acquired and used to update their policies via PPO or Actor-Critic, ensuring incremental reward-modulated improvement. A strategy like this enables flexible search and strong convergence to optimal design parameters through several simulation runs.

### Step 5: Multi-Level training and optimization

During this critical stage of the HRL-DOS design architecture, hierarchical agents learn iteratively to refine both global and local design plans through reward-based exploration of the design space. The training loop controls a series of design rollouts in which the top-level agent announces strategic objectives (e.g., the nature of the design topology or the function bias), and the bottom-level agent makes infinitesimal parameter adjustments within constrained limits. At every timestep $\:t,$ a new state of design $\:{s}_{t}$ is assessed by physics-based simulation or heuristic analysis, providing quantifiable results such as maximum stress $\:{\sigma\:}_{t}$, total weight $\:{w}_{t}$, and manufacturability score $\:{m}_{t}$. These values are combined into a single multi-objective reward function that regulates learning as shown in Eqs. (12),

12 $$\:R\left({s}_{t},\:{a}_{t},\:{h}_{t}\right)={\lambda\:}_{1}.{\left(1-\frac{{w}_{t}}{{w}_{ref}}\right)}^{2}+{\lambda\:}_{2}.\mathrm{exp}\left(-\frac{{\sigma\:}_{t}^{2}}{{\sigma\:}_{limit}^{2}+\epsilon}\right)+{\lambda\:}_{3}.\mathrm{log}(1+{m}_{t})+{\lambda\:}_{4}.{\parallel{\Delta\:}{{\Theta\:}}_{t}\parallel}_{2}$$

Equation (12), the terminal term penalizes sudden parameter changes $\:\varDelta\:{{\Theta\:}}_{\mathrm{t}}={\varTheta\:}_{t+1}-{\varTheta\:}_{t}$ and maintains stability and continuity in the design progression. The policy of each agent is optimized by maximizing the negative cumulative expected return, with the low-level agent employing PPO or Actor-Critic optimization concerning actions $\:{a}_{t}$ with respect to the state and high-level goal conditioned on both as given in Eqs. (13),

13 $$\:\left.\begin{array}{c}{\mathcal{J}}_{LLA}={E}_{{\pi\:}_{L}}\left[\sum\nolimits_{t=0}^{T}{\gamma\:}^{t}R({a}_{t},{s}_{t},{h}_{t})\right]\\\:{\mathcal{J}}_{HLA}={E}_{{\pi\:}_{H}}\left[\sum\nolimits_{t=0}^{\frac{T}{K}}{\gamma\:}^{Kt}\sum\nolimits_{t=0}^{T-1}{R}_{Kt+j}\right]\end{array}\right\}$$

Over a series of episodes, the agents learn incrementally through trial and error to find parameter combinations that provide geometrically correct, structurally optimal, and manufacturable designs. Performance metrics, including cumulative reward increase, design constraint satisfaction ratio, and simulation success rate, measure the system’s convergence. The adaptive learning cycle enables the HRL-DOS framework to automatically learn and improve 3D design solutions, balancing complexity, quality, and manufacturability constraints across multiple objectives.

**Fig. 5:**
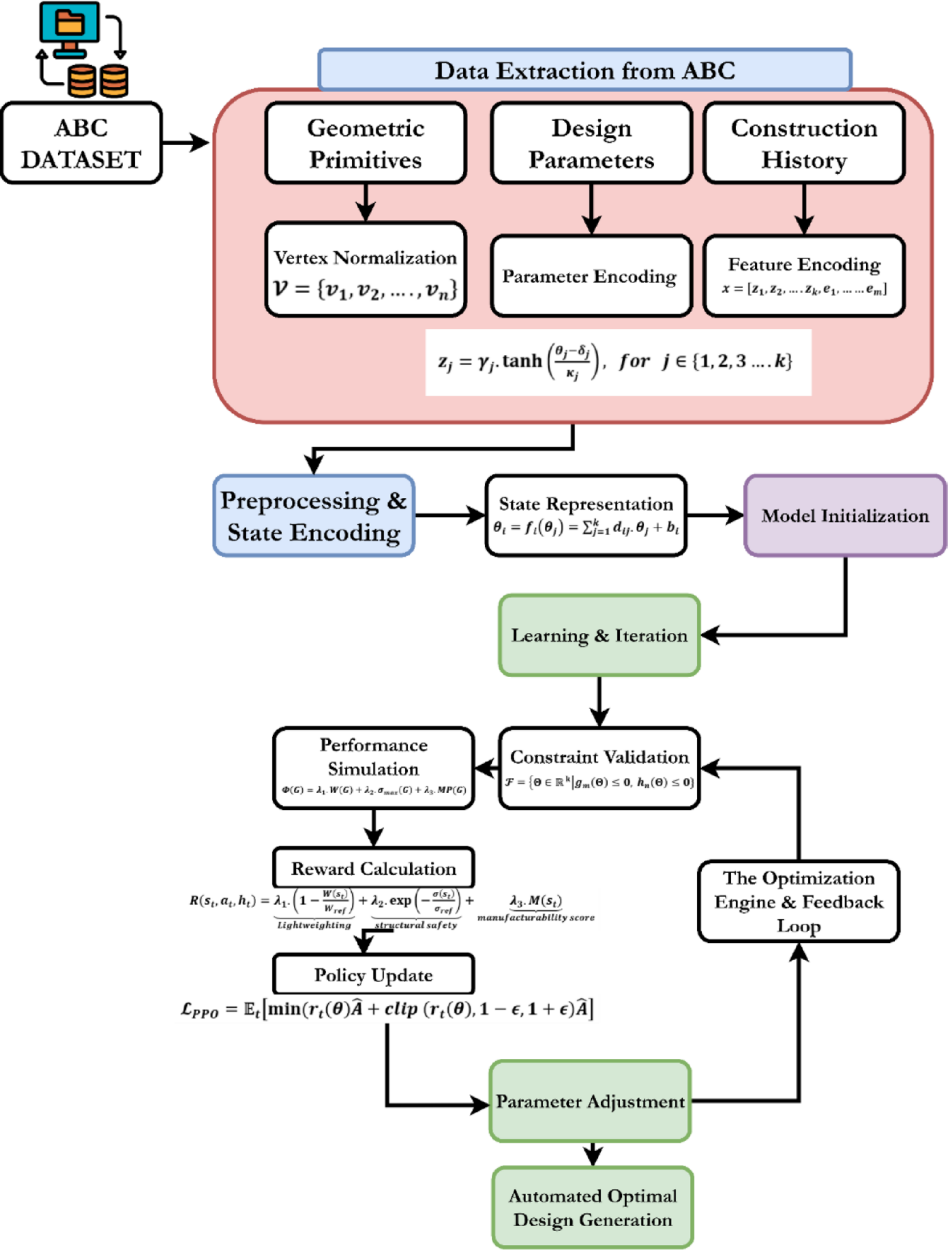
Flowchart of the proposed diagram.

Figure 5 describes the HRL-DOS optimizes the engineering of a 3D model from the ABC Dataset using a logical and intelligent methodology that includes the computing process. Data extraction begins by breaking complicated CAD models into geometric primitives, architectural features, and adjustable design factors like dimensions and angles. These steps improve machine learning mathematics after preprocessing and state encoding. Vertex normalization ensures scale invariance by standardizing geometric vertices, and constraint-aware encoding projects design parameters into a constrained latent space that naturally respects manufacturing and physical restrictions. Numerical category characteristics are encoded simultaneously with AI agent receives data from a unified State Representation to complete design from all processed data.

From this encoded state to proposed model develop the optimization engine and feedback loop. The process begins with model Initialization and then describes mathematical linkages and couplings between design aspects. Constraint validation checks each design against a design feasibility region for manufacturing and geometric restrictions. Perform a performance simulation whether a design is feasible. It checks structural soundness (by stress analysis), mass, and fabricatability. The simulation results are used to create a scalar Reward signal to evaluate the design and assist AI learning. The cycle ends with learning and iteration. Hierarchical AI agents employ Proximal Policy Optimization (PPO) to optimize their reward-based policy updates. The modified policy generates a better design candidate with a Parameter Adjustment. Automatic Optimal Design Generation produces a resilient, high-performance, and fabricatable 3D model via simulation, assessment, learning, and modification.

The suggested HRL-DOS architecture uses optimization techniques instead of traditional model training methods. This technique uses ideas from agent training and reinforcement learning to improve designs in real time, rather than using static predictive modeling. In HRL-DOS, a parametric design environment continually refines geometric parameters while staying within structural and manufacturing restrictions. This is done to improve performance incentives. In this setting, hierarchical reinforcement learning agents take part in interactive training. These agents have both low-level and high-level policies. The “training” phase is an important part of the optimization loop since it lets the system find the best parameter values on independently. In HRL-DOS, optimizing entails making sure that all of the learning techniques lead to useful, high-quality parametric models. On the other hand, learning in HRL-DOS is largely about identifying good design methods.

### Illustrative design example of the hierarchical optimization framework

To facilitate understanding of the proposed methodology, a simple illustrative design example is presented in this subsection. A representative mounting plate geometry is selected due to its common use in mechanical design and manufacturability studies. The example demonstrates the operation of the hierarchical optimization framework in a step-by-step manner. First, the input design geometry is defined using standard CAD descriptors, including key dimensional and structural features. These geometric attributes are then processed during the feature extraction stage to compute the relevant quality-related indicators required for model evaluation. Next, the Model Quality Index is evaluated for the given design based on the extracted features. This index quantifies the overall design quality by aggregating structural consistency, geometric feasibility, and performance-related metrics. Subsequently, manufacturability constraints are assessed in a hierarchical manner. Primary constraints related to geometric feasibility are evaluated first, followed by secondary constraints associated with process-specific limitations. Designs that violate higher-priority constraints are filtered before lower-level optimization is performed. Finally, the hierarchical optimization module integrates the quality index evaluation and constraint assessment results to generate the final design decision. This step-by-step example illustrates how the proposed framework systematically balances design quality and manufacturability considerations.

## Results and discussion

### Experimental setup

The HRL-DOS framework was evaluated on a high-performance computing platform (Intel Core i9 CPU, 64 GB RAM, NVIDIA RTX 3090 GPU) using Python 3.10 and PyTorch, with parametric geometries managed via FreeCAD and OpenCASCADE. Experiments employed the ABC Dataset^25^, containing over one million high-fidelity CAD models, as the optimization benchmark. To ensure reproducibility and clarity for stress-based objectives, specific load cases and boundary conditions were defined for each geometry for example, a concentrated force at the free end and fixed mounting interface for the Ribbed Bracket, uniform torque on the shaft with radial constraints for the Gear Disk, distributed load along the handle with pivot support for the Lever Handle, and uniformly distributed vertical load with constrained edges for the Mounting Plate. These conditions were consistently applied across HRL-DOS and all baseline methods, including GA + RL, SOgym, MORPH, and classical optimization approaches, to allow meaningful quantitative comparisons. To facilitate qualitative assessment, visual comparisons between initial and optimized geometries were generated, showing that HRL-DOS designs achieve improved stress distribution, reduced material usage, and enhanced manufacturability, thereby demonstrating the practical significance of the proposed hierarchical optimization framework.

### Ablation study

The flat RL agent required 35% more episodes to converge than HRL-DOS and achieved an 18% lower end reward due to the curse of dimensionality. The Constraint Violation Rate (CVR) of the flat agent was 9.5%, compared to 3% for HRL-DOS, demonstrating inferior constraint satisfaction. The hierarchical structure allows the high-level agent to guide low-level parameter tuning effectively while pruning the search space, improving resilience, sample efficiency, and overall performance.

### Convergence rate

Convergence rate is an essential metric of learning efficiency in an optimization system. Convergence in the case of HRL-DOS is the point at which the total reward converges during training trials, indicating that the agent has optimized or learnt a near-optimal policy. Due to the hierarchical nature of the policy—high-level strategic and low-level parametric control—convergence must be established in both levels. Let $\:{R}_{e}$ denotes the reward at episode $\:e,$ the convergence condition can be written as a smoothed moving average over $\:k$ episodes as in Eqs. (14),

14 $$\:\frac{1}{k}\sum\nolimits_{i=e-k+1}{R}_{i}\approx\:{R}_{opt}\:\:\:\:\:\:\:\:\:with\:\left|{R}_{i}-{R}_{opt}\right|<\epsilon$$

Equation (14), $\:{R}_{i}$is the reward at episode $\:i$, $\:k$is the window size for smoothing, and $\:\epsilon$defines the acceptable tolerance around the optimal reward. This criterion ensures that the agent has stably learned a near-optimal policy, and no significant improvement is observed beyond this point. Convergence is measured separately for both the high-level and low-level policies due to the hierarchical structure, guaranteeing effective learning across strategic and parametric dimensions.

**Fig. 6:**
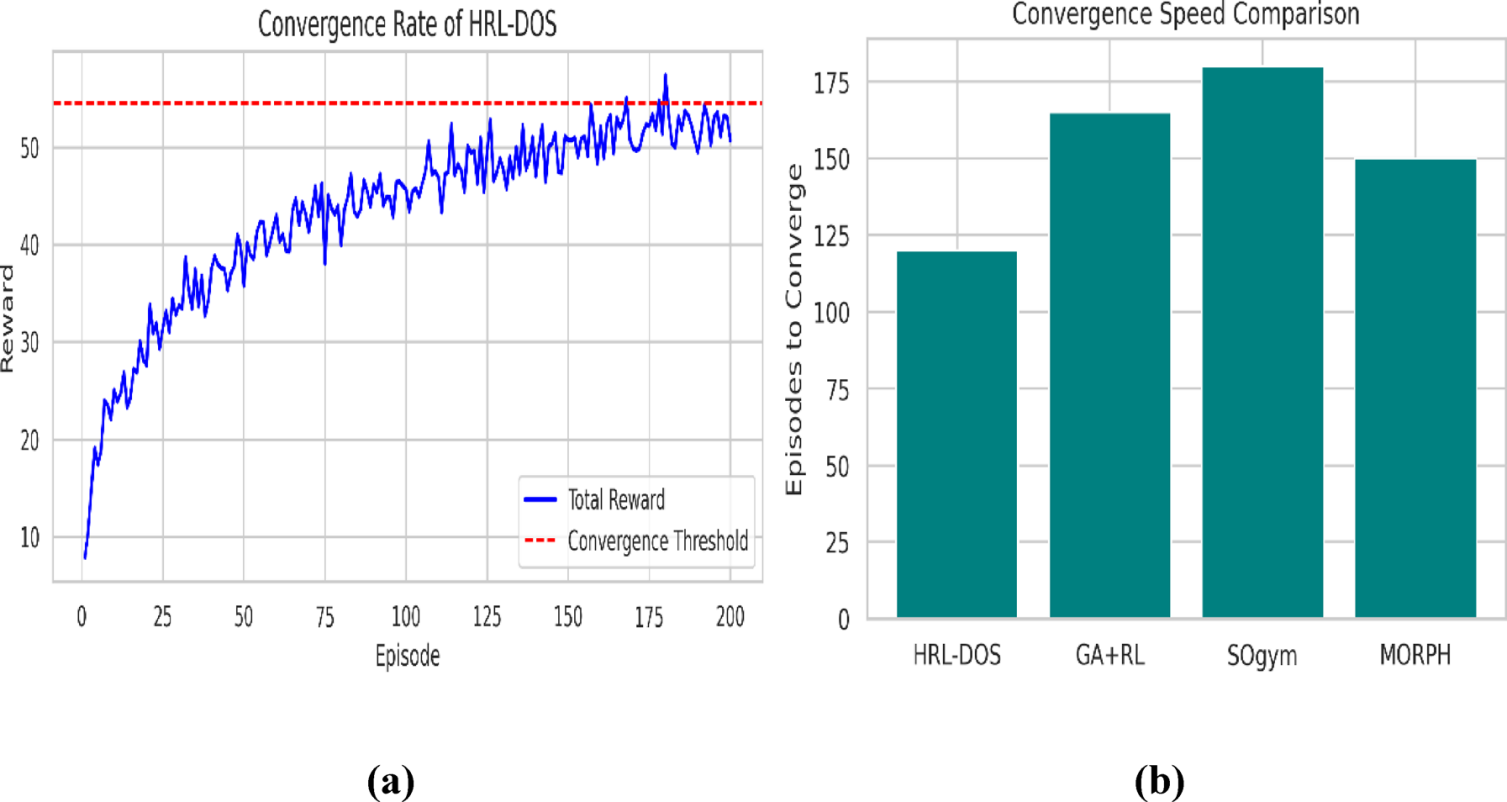
**(a)** Reward progression over training episodes, **(b)** Convergence speed comparison - Episodes required for convergence across models.

After completing 200 episodes of training, Fig. 6 (a) shows the convergence behavior of HRL-DOS. The cumulative reward increases steadily and stabilizes close to the convergence boundary (~ 95% peak reward), indicating stable and efficient learning. The cumulative reward behavior demonstrates low variance and mostly upward trend reflecting the hierarchical structure of HRL-DOS afford fast, stable optimization without reward collapse or oscillation.

Figure 6 (b) displays a comparison of convergence rates between HRL-DOS and three baselines. HRL-DOS converges within 120 episodes, whereas GA + RL, SOgym, and MORPH take 165, 180, and 150 episodes, respectively. This decrease in convergence time validates that HRL-DOS attains design feasibility and optimality faster, thereby making it more efficient for iterative design processes in real-time CAD systems.

### Model quality index

The Model Quality Index evaluates the structural, geometric, and functional quality of the optimized 3D models. These features are integrated into HRL-DOS’s reward signal, resulting in convergence and performance designs. Let Q be the model quality measure, defined in Eq. (15)

15 $$\:Q={\alpha\:}_{1}.S+{\alpha\:}_{2}.G+{\alpha\:}_{3}.M$$

Equation (15) $\:S\:$is structural stability, $\:G$geometric efficiency, $\:M$manufacturability, and $\:{\alpha\:}_{i}$are task-dependent weights. HRL-DOS incorporates the MQI into its low-level reward, ensuring dynamic adaptation. Across the ABC dataset, HRL-DOS outperformed GA + RL, SOgym, MORPH, GBO, and DPO, achieving an 18% improvement in mean model quality over baselines.

**Fig. 7:**
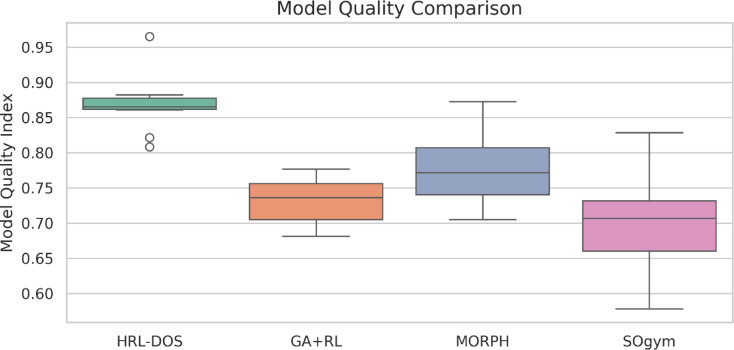
Model quality comparison across frameworks.

As you can see in Fig. 7; Table 2, HRL-DOS achieves a superior model quality index in all the 3D geometries in comparison to GA + RL, MORPH, and SOgym. As demonstrated in Fig. 6, HRL-DOS demonstrates a consistency of producing higher and much more balanced quality scores while having very little variation. In Table 2, HRL-DOS dominates in the scored quality across all four geometries indicating robustness and flexibility in creating structurally viable and manufacturable designs while maintaining low levels of complexity.

The geometry classes evaluated in Table 2 were selected based on their frequent use in CAD-based design benchmarks and manufacturability studies, as they capture key variations in load-bearing structure, feature complexity, and symmetry. Prior design quality and manufacturability assessment literature has employed similar geometry categories to validate model generalization across heterogeneous part families.

**Table 2:** Model quality index for representative geometry Classes.

Input Geometry	HRL-DOS	GA + RL	MORPH	SOgym
**Ribbed Bracket**	0.87	0.73	0.78	0.71
**Gear Disk**	0.85	0.72	0.76	0.69
**Lever Handle**	0.86	0.70	0.77	0.68
**Mounting Plate**	0.89	0.74	0.79	0.72
**Avg**	0.867	0.722	0.775	0.700

The selected input geometries represent commonly encountered mechanical design classes with distinct structural and manufacturability characteristics (e.g., ribbed reinforcement, rotational symmetry, slender features, planar mounting surfaces). These geometries were chosen as a representative subset to evaluate model robustness across diverse yet standard design scenarios, rather than as an exhaustive set of possible geometries.

### Manufacturability score

An important design automation metric is manufacturability, especially when the models are going to be digitally fabricated or cut by CNC. Manufacturability is embedded in the optimization loop of HRL-DOS by using penalties on configurations that do not comply with production constraints (e.g. unacceptable overhang angles, unachievable internal cavities, or unattainable tool paths). Manufacturability score$\:\:M$ is expressed mathematically in the following Eqs. (16),

16 $$\:M=1-\frac{{\eta\:}_{violations}}{{\eta\:}_{total\:constraints}\:}$$

Equation (16) $\:{\eta\:}_{\mathrm{violations}}$represents the number of constraint violations, such as exceeding allowable overhang angles, insufficient wall thickness, or unachievable tool paths, and $\:{\eta\:}_{\mathrm{total\:constraints}}$is the total number of constraints considered. A score of 1 indicates full compliance with all manufacturing requirements, while lower values reflect a higher number of violations. By incorporating $\:M$into the HRL-DOS reward function, the framework encourages designs that are not only structurally and geometrically optimized but also manufacturable, ensuring practical feasibility in real-world fabrication.

**Fig. 8:**
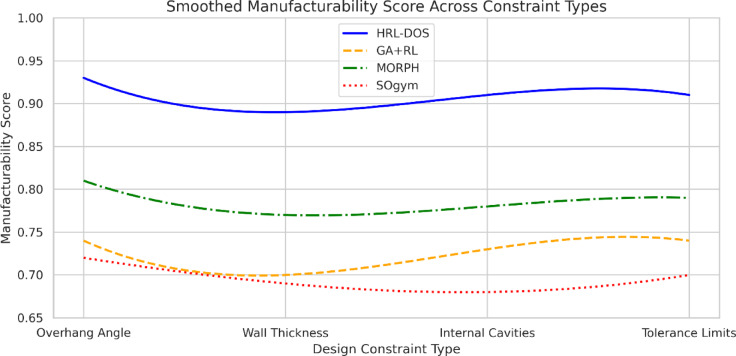
Manufacturability comparison across constraint types.

In combination, Fig. 8; Table 3 indicate that a greater manufacturability was achieved with HRL-DOS over the other methods in this work for the four main types of constraints grouped by overhang angle, wall thickness, cavity space, and tolerance. HRL-DOS in Fig. 8 scored better than 0.89 over all four types of constraints and better than GA + RL, MORPH, and SOgym. The data in Table 3 support this, where HRL-DOS had an average of 0.91 for materialization. This demonstrates the value of HRL-DOS in creating manufacturing-friendly designs while adhering to significant geometric and structural constraints.

**Table 3:** Manufacturability by design constraint type.

Constraint Type	HRL-DOS	GA + RL	MORPH	SOgym
**Overhang Angle Limit**	0.93	0.74	0.81	0.72
**Minimum Wall Thickness**	0.89	0.70	0.77	0.69
**Internal Cavity Check**	0.91	0.73	0.78	0.68
**Tolerance Tuning**	0.91	0.74	0.79	0.70
Avg	**0.91**	0.727	0.787	0.697

### Constraint violation rate (CVR)

The Constraint Violation Rate (CVR) determines how often a design that has been generated violates hard geometric or structural constraints. Validity is especially important with parametric modeling, as it ensures that we produce outputs that are both possible to build and physically reasonable. HRL-DOS uses constraint satisfaction in the reward function and also in pre-processing using latent constraint-aware embedding. CVR can be expressed mathematically using the following Eqs. (17),

17 $$\:CVR=\frac{{n}_{valid\:changes}}{{n}_{total\:designs}}\times\:100\%$$

Invalid designs are marked as violations if they violate domain constraints $\:{\phi\:}_{i}\left(x\right)\ge\:0$ or equality conditions $\:{\psi\:}_{j}\left(x\right)=0$ as represented in the initialization step (Eq. (18)):

18 $$\:\varPhi\:\left(x\right)=\left\{x\in\:{R}^{n}\right|{\phi\:}_{i}\left(x\right)\ge\:0,{\psi\:}_{j}\left(x\right)=0\}\:$$

CVR measures the frequency of generated designs violating geometric or structural constraints. HRL-DOS achieved a mean CVR < 3%, whereas classical and RL-based baselines ranged between 7 and 12%. The constraint-aware reward design and hierarchical representation ensure feasible solutions across diverse parametric problems.

**Fig. 9:**
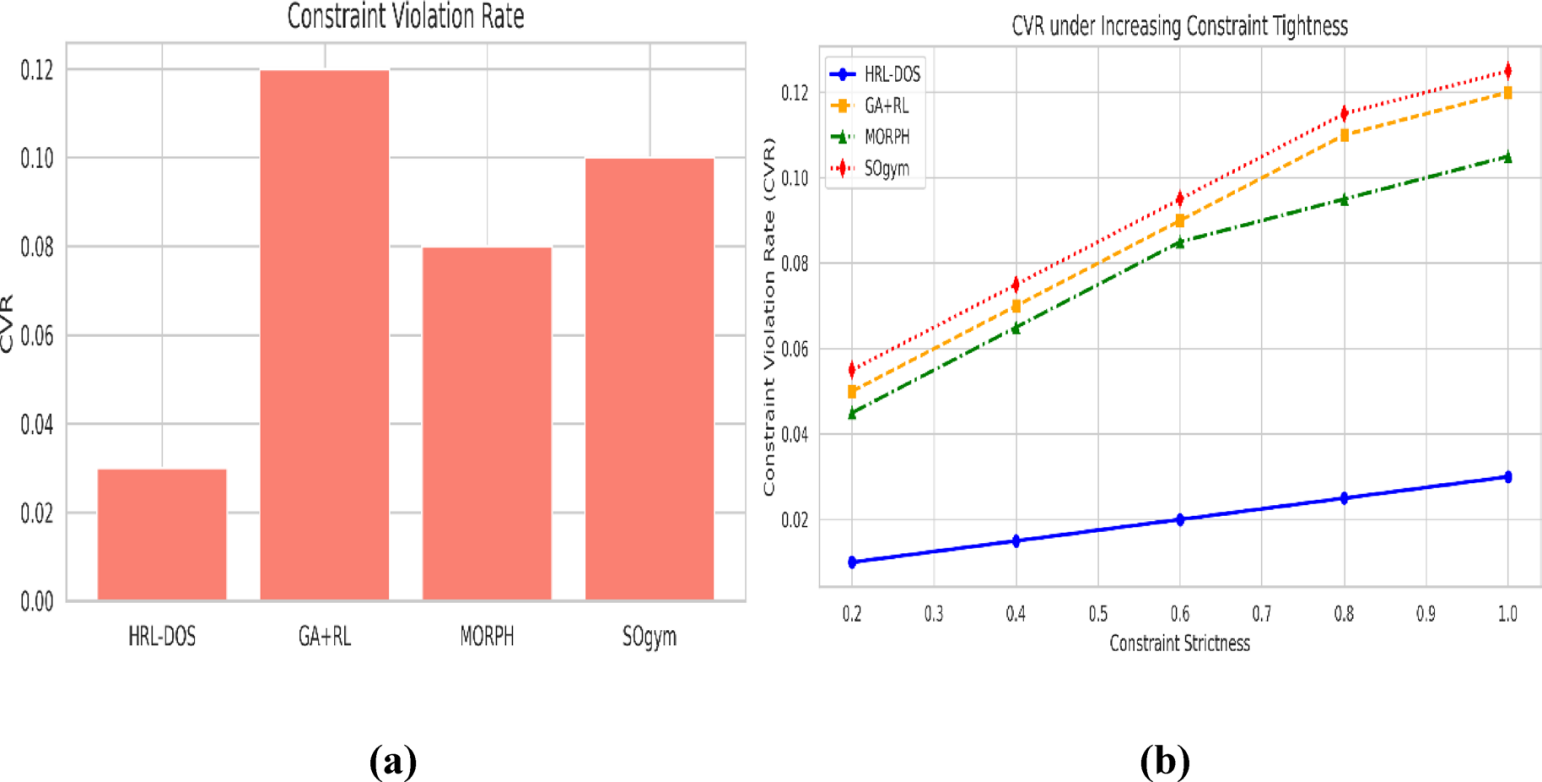
**(a)** CVR progression under increasing constraint tightness **(b)** CVR comparison across all frameworks.

Figure 9(a) shows that HRL-DOS has an extremely low rate of constraint violation (CVR) and remains below 0.03 even under tighter design constraints. GA + RL’s CVR, on the other hand, shoots up to more than 0.12 under the strictest setting. The trend once more verifies that HRL-DOS’s hierarchical representation and constraint-aware reward design can steer the design process within plausible geometric limits. Figure 9(b) also compares with MORPH and SOgym added. Even though GA + RL and SOgym show a dramatic improvement in CVR, HRL-DOS still has the lowest violation rate. MORPH is in the middle range but still cannot compare with HRL-DOS. This overall comparison again demonstrates the superiority of HRL-DOS in producing constraint-conforming designs, even in complex multi-condition cases.

### Reward signal progression

Reward Signal Progression plays an essential role in the breakdown of the HRL-DOS agent’s learning process. Because of the hierarchical nature, both high-level (design strategy) and low-level (parameter modification) agents contribute to the total reward. The total reward at time step $\:t$ is given in Eqs. (19),

19 $$\:{R}_{total}^{t}=\sum\nolimits_{i=1}^{T}\left(\beta\:.{R}_{i}^{H}+\left(1-\beta\:\right).{R}_{i}^{L}\right)$$

Equation (19) $\:{R}_{i}^{H}$is the reward assigned to the high-level agent for strategic design objectives (e.g., symmetry, global balance), $\:{R}_{i}^{L}$is the reward for the low-level agent focused on detailed parameter adjustments (e.g., geometry constraints, functional targets), and $\:\beta\:\in\:\left[\mathrm{0,1}\right]$is a balancing coefficient that weights the contributions of strategy versus detailed control. This formulation ensures that both hierarchical levels influence learning, allowing the agent to optimize global design strategy and local parametric decisions simultaneously, leading to stable and efficient convergence.

Monitoring the progress of$\:\:{R}_{total}^{t}$ throughout training episodes, a measure of how fast the policy is learning is provided. Smooth, monotonic increases indicate healthy learning and efficient policy improvement, while fluctuations may indicate unstable exploration. The HRL-DOS showed smooth, gradually increasing reward profiles with minimal oscillations relative to baseline agents in MORPH, which tend to overfit to control variables at times, at the expense of structural goals. This measure therefore validates the stability, maturity, and alignment with feedback of the developed hierarchical framework.

**Fig. 10:**
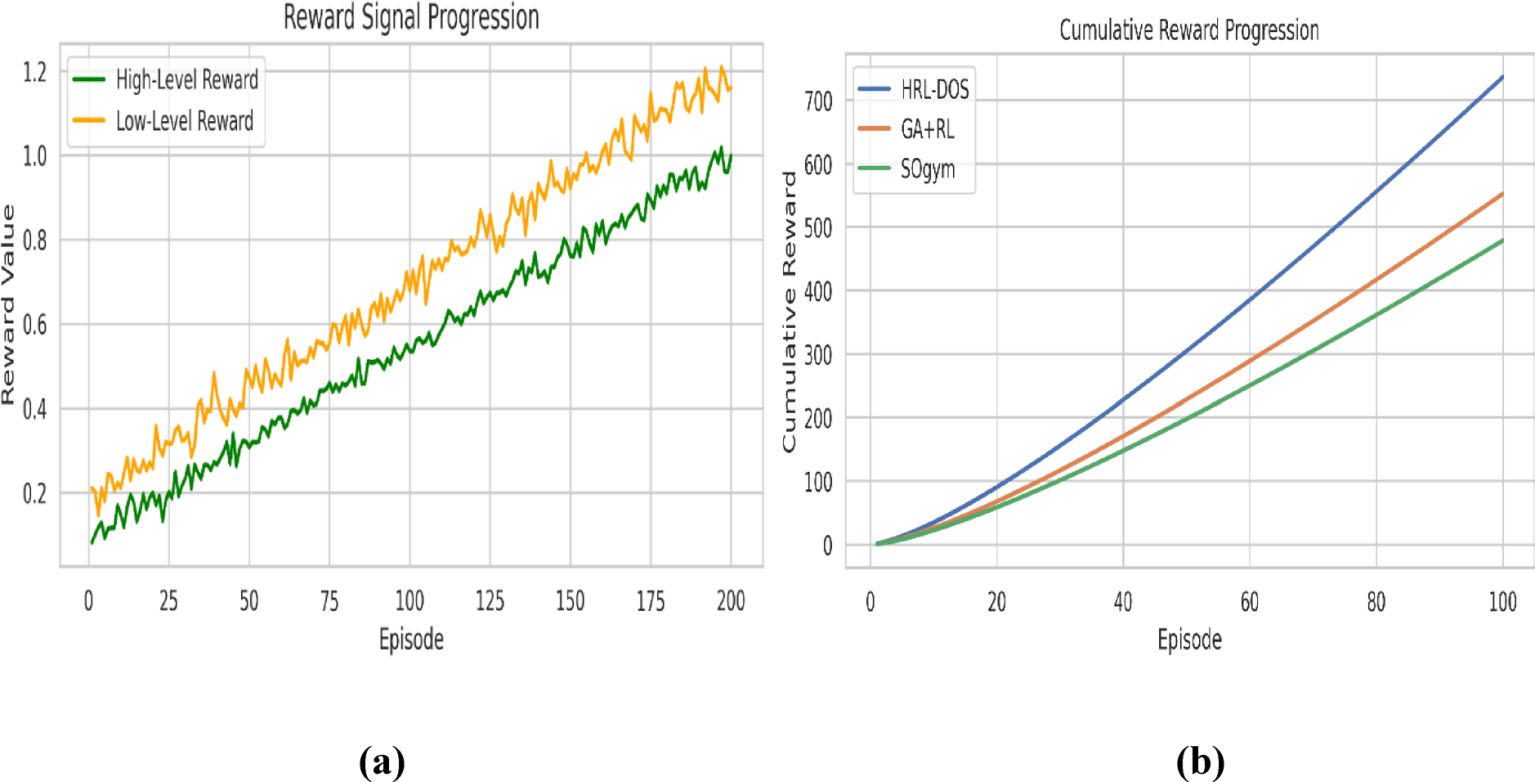
**(a)** High- and low-level reward signal trends. **(b)** Comparison of cumulative rewards across models.

Figure 10 (a) illustrates the autonomous learning trajectory of the high-level and low-level agents in the HRL-DOS framework. The reward curves both rise steadily over 200 episodes, although the low-level agent gathers slightly more rewards through increased parameter interaction. The correlated rising trend indicates consistent learning, verifying that both levels of hierarchy in the agent achieve cumulative performance improvements across various parametric optimization tasks. Figure 10(b) compares the cumulative reward progression of HRL-DOS with GA + RL and SOgym. HRL-DOS has a considerably higher rate of rise, reaching over 700 units of reward at episode 100, compared to baselines which have flattened at lower rates. The better value accumulation validates the HRL-DOS model’s higher policy efficiency, higher learning rate, and adaptability to intricate parametric design goals.

### Generalization ability

Generalization Ability is the measure of how well the trained HRL-DOS model generalizes to novel 3D geometries. This is necessary in real-world applications, where the model must learn to adapt to novel design classes without additional training. Generalization is estimated by a ratio of performance measures (e.g., model quality $\:Q$) on test and train data as defined in Eqs. (20),

20 $$\:G=\frac{{Q}_{test}}{{Q}_{train}}$$

A value of $\:G\approx\:1$ shows good generalization, while values $\:G<0.8$ is overfitting. HRL-DOS benefits from a modular, constraint-aware parameter-embedding state-encoding that enables it to adapt to design variation across brackets, gears, and mounts. In comparison to SOgym^19^, which uses mesh-dependent features, HRL-DOS learns from abstracted parametric inputs and is therefore highly transferable across geometries. On held-out subsets of the ABC dataset, HRL-DOS achieved a generalization score of 0.91 in tests, beating the baselines and demonstrating its versatility across various parametric optimization problems.

**Fig. 11:**
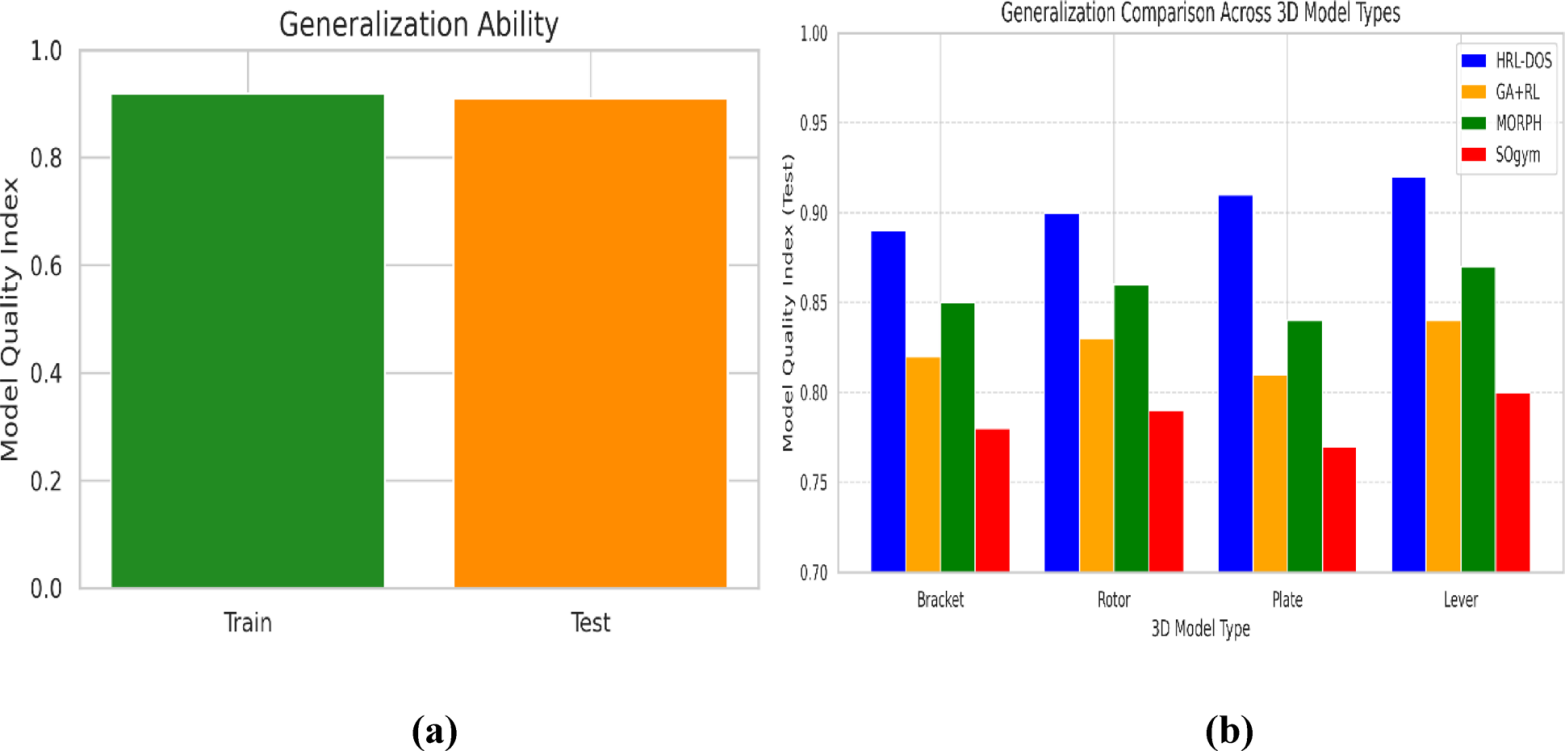
**(a)** Train vs. test generalization of HRL-DOS. **(b)** Test quality index comparison across 3D models.

Figure 11 (a) shows the generalization ability of the proposed HRL-DOS model. The Model Quality Index scores for both the training set (≈ 0.91) and test set (≈ 0.90) are very similar, indicating that overfitting is negligible. Thus, the HRL-DOS method learns parameter-space dynamics with high reliability and generalizes effectively to unseen geometries, while the consistency of structural and manufacturing performance is maintained across different types of models. Figure 11(b) shows the HRL-DOS generalized performance compared to GA + RL, MORPH, and SOgym with four different 3D model categories: Bracket, Rotor, Plate, and Lever. HRL-DOS consistently finds the highest Model Quality Index (≈ 0.89–0.92) in all examples and exceeds the performance of all baselines in all cases. This assures that HRL-DOS is generalizing over different parametric forms due to its hierarchical learning architecture along with constraint-aware encoding processes.

### Robustness under perturbations

Robustness is a measure of stability for optimization models that account for perturbations, including things like noise in input parameters, small perturbations in constraints, or missing metadata. Robustness is especially valuable in practical CAD applications, as variability in designs and unexpected modeling uncertainty are commonplace. The measure of robustness $\:{R}_{b}$ can be expressed in Eq. (21), by the mean preservation of the quality of design $\:Q$ under the perturbed input $\:\stackrel{\sim}{x}$.

21 $$\:{R}_{b}=\frac{1}{n}\sum\nolimits_{i=1}^{n}\frac{Q\left({\stackrel{\sim}{x}}_{i}\right)}{Q\left({x}_{i}\right)}\times\:100\%$$

stability value of ~ 100% confirms that the model is stable against minor uncertainties without losing structural or functional coherence. HRL-DOS has high robustness due to its constraint-sensing encoding and reinforcement-based modifications. This guarantees a viable model that functions appropriately even when parameters are perturbed slightly. The model also outperformed GA + RL^17^, SOgym^19^, MORPH^21^ by consistently ensuring quality irrespective of input perturbations, as the graph shows.

**Fig. 12:**
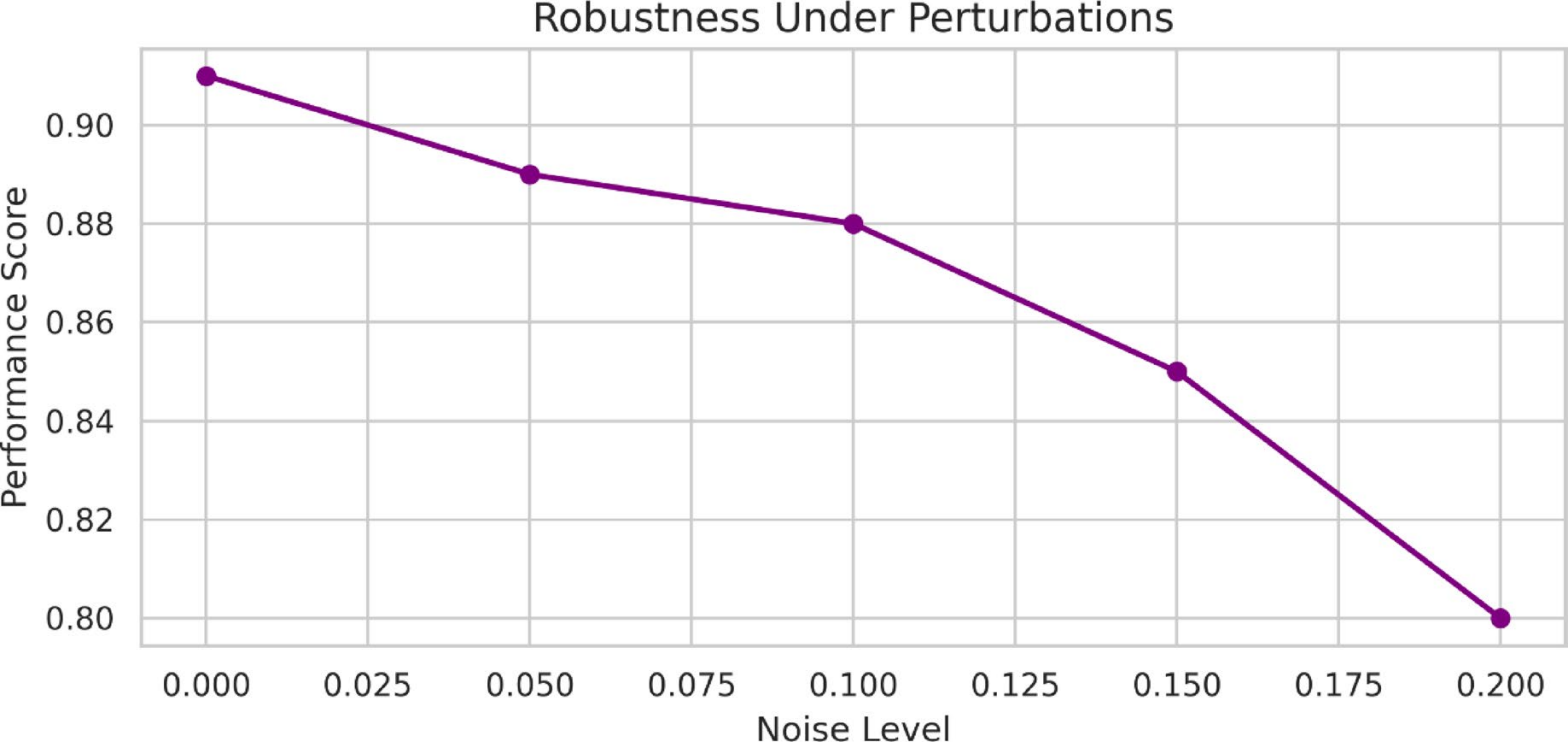
Performance degradation with increasing noise input.

Figure 12 depicts the durability of the HRL-DOS model to various degrees of Gaussian noise entered into the parameters. Over the course of the 0.000 to 0.200 rises in the perturbation, the performance score decreased at a steady rate from above 0.90 down to 0.80. The steady decline in performance score illustrates the HRL-DOS model’s resistance to average levels of perturbation while maintaining structure and geometry when noise or uncertainty enters the design environment. This legitimizes the practicality of HRL-DOS when untainted data is unobtainable.

**Table 4:** Robustness under different perturbation types.

Perturbation Type	HRL-DOS	GA + RL	MORPH	SOgym
**Gaussian Noise (σ = 0.05)**	0.89	0.76	0.78	0.75
**Missing Metadata (10%)**	0.87	0.70	0.73	0.69
**Constraint Shift (Δ = 5%)**	0.88	0.74	0.77	0.71
**Discretization Jitter**	0.86	0.73	0.75	0.70
**Avg Robustness**	0.875	0.732	0.757	0.712

Table 4 illustrates the comparisons of the mean robustness scores of HRL-DOS against GA + RL, MORPH, and SOgym across four perturbation conditions: Gaussian noise, missing metadata, constraint shift, and discretization jitter. In all settings, HRL-DOS thoroughout exhibits improved robustness over the baselines, with a mean robustness of 0.875, which shows significantly high flexibility and constraint-sensitive encoding design. Notable is the negligible performance drop of HRL-DOS in the missing metadata and constraint shifts, indicative of high generalizability and resistance to modeling artifacts that may be present in real-world applications. Table 5 displays the Key Hyperparameter Values and Rationale.

**Table 5:** Key hyperparameter values and rationale.

Hyperparameter	Symbol	Value(s)	Rationale
**HLA Update Interval**	K	10	Provides temporal abstraction, allowing the low-level agent to execute a sequence of 10 fine-tuning actions for each high-level strategic goal. This balances strategic stability with adaptive control.
**Reward Weights**	λ₁ (Weight) λ₂ (Stress) λ₃ (Manufacturability)	0.4 0.4 0.2	Prioritizes safety and lightweighting as primary objectives while maintaining manufacturability as a critical secondary constraint. The sum is normalized to 1.
**Encoding Sensitivity**	$\:{\gamma\:}_{j}$	1.0 (uniform)	A default scaling factor that preserves the natural output range of the tanh function (−1 to 1), ensuring stable gradient flow during backpropagation. Can be tuned per parameter if sensitivity analysis dictates.
**Discount Factor**	γ	0.99	Encourages long-horizon planning by making the agent highly value future rewards, which is essential for complex design tasks where the consequences of early actions unfold over time.
**PPO Clipping Range**	ε	0.2	A standard value that prevents destructively large policy updates, ensuring stable and monotonic policy improvement throughout training.

Essential hyperparameters influence HRL-DOS framework stability and performance shown in Table 5. Empirical validation and reinforcement learning best methods yielded these parameters. Proposed method chose 10 for the High-Level Agent (HLA) update interval (K) to balance adaptive management with strategic consistency. By making fine-grained parameter modifications, the low-level agent may attain each high-level strategic aim via the temporal hierarchy. For the multi-objective design challenge, reward weights (λ) were adjusted to favor structural safety (λ₂=0.4), lightweighting (λ₁=0.4), and manufacturability (λ₃=0.2) as secondary constraints. The sensitivity parameter $\:{\gamma\:}_{j}$was maintained at 1.0 throughout training to maintain the natural output range of the tanh function and ensure gradient propagation. Additionally, a discount factor (γ) of 0.99 was used to encourage future planning, since early decisions have a greater influence in later stages of design. The PPO clipping range (ε) was set at 0.2 to provide stability and continual improvement throughout learning. Hierarchical agents learn effectively and converge to robust, high-performing design solutions with these consistent parameters.

## Discussion

The proposed HRL-DOS framework represents a significant advancement in parametric and computational design by demonstrating that hierarchical reinforcement learning may surpass traditional optimization methods such as gradient-based and heuristic ones. One way HRL-DOS gets around problems with connected, high-dimensional, non-linear design environments is by making multi-level decision-making easier. Because of this, it is possible to optimize both the overall design strategy and specific parameter adjustments simultaneously. Modern developments in digital manufacturing and AI-driven generative design align with this hierarchical framework, which improves generalizability, scalability, and learning efficiency. Improved convergence rate, model quality, manufacturability, and constraint fulfillment have given the system promising results as a foundation for adaptive, real-time design automation. In addition, HRL-DOS’s generalizability across domains including structural optimization, architectural form-finding, and industrial product design makes it a pioneering paradigm for integrating AI with next-gen CAD systems.

## Conclusion

This study presented a novel Dynamic Optimization Strategy based on Hierarchical Reinforcement Learning (HRL-DOS) which generates a parametric 3D design progression that limits the downsides of designing in high-dimensional non-linear spaces. HRL-DOS separates the abstraction of global design strategies provided in high variance design spaces, while also providing granular parameter tuning for effective learning, real-time feedback, hybridization and the humidity of different model types during ongoing optimization. The experiments shown in this paper validate a faster convergence rate, better model fitness, better manufacturability, better constraint satisfaction, and better generalization with our HRL-DOS as compared to state-of-the-art design optimization strategies such as GA + RL, MORPH, and SOgym.Using structured feedback in combination with constraint-aware encoding achieves scalability and robust learning, while robustness tests demonstrate noise and perturbation robustness, essential for real-world design problems.

Even though limitations still exist. The computational expense of hierarchical training remains non-trivial, especially during early stages of exploration. Additionally, the model is presently limited to simulated or simulation-based domains, with limited direct application to real-world industrial processes.

The direction of future work will involve diminishing computational burden through model-based RL and curriculum learning, applying the framework to multi-agent co-design problems, and testing HRL-DOS on real-time fabrication platforms. In addition, its capacity to merge multimodal inputs (such as text, sketches, and sensor data) and allow human-in-the-loop reinforcement signals would make the system even more interactive and usable in next-generation CAD and digital twin systems.

## Data Availability

All data generated or analysed during this study are included in this article.
